# Tumor Cell–Autonomous SHP2 Contributes to Immune Suppression in Metastatic Breast Cancer

**DOI:** 10.1158/2767-9764.CRC-22-0117

**Published:** 2022-10-03

**Authors:** Hao Chen, Gregory M. Cresswell, Sarah Libring, Mitchell G. Ayers, Jinmin Miao, Zhong-Yin Zhang, Luis Solorio, Timothy L. Ratliff, Michael K. Wendt

**Affiliations:** 1 Department of Medicinal Chemistry and Molecular Pharmacology, Purdue University, West Lafayette, Indiana.; 2 Department of Comparative Pathobiology, Purdue University, West Lafayette, Indiana.; 3 Department of Biomedical Engineering, Purdue University, West Lafayette, Indiana.; 4 Purdue University Center for Cancer Research, Purdue University, West Lafayette, Indiana.

## Abstract

SH2 containing protein tyrosine phosphatase-2 (SHP2) is recognized as a druggable oncogenic phosphatase that is expressed in both tumor cells and immune cells. How tumor cell–autonomous SHP2 contributes to an immunosuppressive tumor microenvironment (TME) and therapeutic failure of immune checkpoint blockades in metastatic breast cancer (MBC) is not fully understood. Herein, we utilized systemic SHP2 inhibition and inducible genetic depletion of SHP2 to investigate immune reprogramming during SHP2 targeting. Pharmacologic inhibition of SHP2 sensitized MBC cells growing in the lung to α-programmed death ligand 1 (α-PD-L1) antibody treatment via relieving T-cell exhaustion induced by checkpoint blockade. Tumor cell–specific depletion of SHP2 similarly reduced pulmonary metastasis and also relieved exhaustion markers on CD8^+^ and CD4^+^ cells. Both systemic SHP2 inhibition and tumor cell–autonomous SHP2 depletion reduced tumor-infiltrated CD4^+^ T cells and M2-polarized tumor-associated macrophages. Analysis of TCGA datasets revealed that phosphorylation of SHP2 is important for immune-cell infiltration, T-cell activation and antigen presentation. To investigate this mechanistically, we conducted *in vitro* T-cell killing assays, which demonstrated that pretreatment of tumor cells with FGF2 and PDGF reduced the cytotoxicity of CD8^+^ T cells in a SHP2-dependent manner. Both growth factor receptor signaling and three-dimensional culture conditions transcriptionally induced PD-L1 via SHP2. Finally, SHP2 inhibition reduced MAPK signaling and enhanced STAT1 signaling, preventing growth factor–mediated suppression of MHC class I. Overall, our findings support the conclusion that tumor cell–autonomous SHP2 is a key signaling node utilized by MBC cells to engage immune-suppressive mechanisms in response to diverse signaling inputs from TME.

**Significance:**

Findings present inhibition of SHP2 as a therapeutic option to limit breast cancer metastasis by promoting antitumor immunity.

## Introduction

Metastatic breast cancer (MBC) is the most advanced stage of breast cancer (stage IV) with lower 5-year survival rates and higher treatment costs than localized disease (1, 2). Cases of MBC are also estimated to increase 54.8% by the end of this decade compared with 2015 (3). Hence, developing novel therapeutic strategies to treat MBC is of immediate clinical importance. Immune checkpoint blockade (ICB) is an important therapeutic in MBC with more than 200 active clinical trials focusing primarily on blockade of programmed cell death protein 1/programmed death-ligand 1 (PD-1/PD-L1) axis (4, 5). However, response to ICB can be difficult, which has led to the recent approval withdrawal of atezolizumab for the treatment of MBC (6, 7). Pembrolizumab has been recently approved in combination with chemotherapy for treatment of early-stage triple-negative breast cancer, but response of this treatment in the metastatic setting is difficult to predict (8, 9).

The tumor microenvironment (TME) is composed of numerous immune cell populations such as CD8^+^ cytotoxic T cells and M2-polarized tumor-associated macrophages (10). These cells have diverse functions that can be modulated in response to different signaling inputs (11). This immune diversity can limit the therapeutic potential of ICB and other targeted therapies in the metastatic setting. Thus, validation of multifunctional therapeutic targets that have the potential to influence the heterogeneous cell populations in the metastatic TME may hold the key to successful application of ICB.

SH2 containing protein tyrosine phosphatase-2 (SHP2) is a promising candidate for a multifunctional therapeutic target as it is a druggable oncogenic phosphatase expressed in both tumor cells and immune cells (12–14). In tumor cells, multiple studies, from our lab and others, have revealed that SHP2 is a key shared node regulating multiple growth factor and survival pathways (15–20). In T cells, SHP2 interacts with immune checkpoints, including PD-1, and inhibits CD28 signaling to induce suppression of T cells (21–25). In addition to lymphocytes, myeloid-specific deletion of SHP2 also suppresses tumor growth *in vivo* (26). Before achieving an active state, a structural alternation is required for SHP2 to release its PTP catalytic domain from auto-inhibitory interaction with its N-SH2 domain (10, 27, 28). Hence, SHP2 can be pharmacologically inhibited by allosteric binders, including SHP099 and TNO155, which stabilize SHP2 in its inactive form (29, 30). Systemic administration of these SHP2 inhibitors showed promising antitumor effects, and some active clinical trials with SHP2 inhibitors have recently emerged (31–34).

Herein we sought to address the hypothesis that tumor cell–autonomous SHP2 contributes to an immune suppressive TME through its regulation of receptor tyrosine kinase (RTK) and extracellular matrix (ECM) signaling. Using a doxycycline-inducible approach, we demonstrate that MBC-cell specific depletion of SHP2 reduces pulmonary metastasis. Mechanistically, inhibition of SHP2 in MBC cells biases upstream signaling toward STAT1 signaling, leading to enhanced expression of MHC class I. Overall, our studies further expand the notion of SHP2 inhibition as a promising strategy to combine with ICB to treat MBC.

## Materials and Methods

### Cell Lines and Cell Culture

The growth conditions of the cell lines in this study are described in Supplementary Table S1. The 4TO7 and D2.A1 cells were obtained from Fred Miller lab at Wayne State University (Detroit, MI). The construction of bioluminescent 4T1 and D2.A1 cells was previously described (35, 36). The other cell lines were purchased from ATCC. All cell lines were authenticated via the IDEXX IMPACT III CellCheck. All cell lines are regularly tested for *Mycoplasma* contamination by PCR.

### Animal Care, Dosing, and Depletion Experiments

All *in vivo* studies were performed in 4-to 6-week old, female BALB/cJ mice purchased from Jackson Laboratories. For the combination study in D2.A1 model, 1  ×  10^6^ cells were injected via the lateral tail vein. The SHP099 was administered via oral gavage, and the α-PD-L1 antibodies were administered via intraperitoneal injection at the indicated concentrations and frequencies. The mice were sacrificed at the end of study, and the tumor-bearing lungs were fixed by 10% formaldehyde overnight. Paraffin tissue sectioning and hematoxylin and eosin (H&E) staining were executed by AML Laboratories, Inc. In the 4T1 spontaneous metastasis model, the 4T1 cells bearing doxycycline-inducible depletion of SHP2 were constructed, sorted and verified as previously described (19). Then, 5  ×  10^4^ cells were engrafted onto the mammary fat pads via an intraductal injection. Doxycycline was administrated in drinking water at 2 mg/mL and refreshed every fourth day following the surgical removal of primary tumors. Reagent manufactures and gavage formulations are listed in Supplementary Table S2. Metastasis in both models was monitored using bioluminescent imaging after intraperitoneal injection of luciferin (GoldBio) using an AMI HT (Spectral Instruments). All *in vivo* studies were performed under IACUC approval from Purdue University (West Lafayette, IN). No randomization or blinding was done.

### Pulmonary Tumor, Spleen Isolation/Digestion, and Flow Cytometry

Tumor bearing lungs were harvested, imaged, weighed, and dissociated with Mouse Tumor Dissociation Kit (Miltenyi Biotec) and GentleMACS Dissociator (Miltenyi Biotec) immediately after sacrificing the mice. The spleens were harvested, weighted, and mechanically disrupted by grinding. The cell suspension was filtered through 70-μm sterile cell strainers and treated with ACK buffer to lyse red blood cells. The single-cell suspension was incubated with TruStain FcX (BioLegend) at 1:50 and Zombie violet (BioLegend) at 1:100. The single-cell suspension from pulmonary tumors was separated into two tubes and subsequently stained with panels of lymphoid antibodies and panels of myeloid antibodies at 1:200 per antibody, respectively. The single-cell suspension from the spleens was subsequently stained with panels of lymphoid antibodies only. Considering the influence of GFP induction with doxycycline induction, the antibody panels were different for the two models. The antibodies for the D2.A1 model and 4T1 model were listed in Supplementary Table S3. The stained cells were fixed with 10% formaldehyde. Within 1 week of staining, flow cytometry was performed using the Fortessa LSR flow cytometry cell analyzer (BD Biosciences). The results were analyzed in a closed-label manner with FlowJo (10.0.7) software.

### Clinical Dataset Analysis and Code Availability

Reverse-phase protein array (RPPA) dataset, mRNA dataset, and clinical outcomes dataset of patients with breast cancer in TCGA were achieved from Firebrowse (http://firebrowse.org/) hosted by Broad Institute by selecting the cohort as “Breast Invasive Carcinoma (BRCA)” on the left panel, and clicking “Reverse Phase Protein Array”, “mRNA” and “Clinical” bars on the right panel. The primary files “RPPA_AnnotateWithGene (MD5)” for RPPA data, “mRNA_Preprocess_Median (MD5)” for mRNA data and “Merge_Clinical (MD5)” for clinical outcomes were downloaded as txt file, and stored locally as raw files named “RPPA_raw.csv”, “mRNA_raw.csv” and “Clinical_raw.csv”.

Immune scores and stromal scores were achieved from an online tool provided by MD Andersen Cancer Center (https://bioinformatics.mdanderson.org/estimate/disease.html) by selecting the “Disease Type” as “Breast Cancer” and the “Platform Type” as “RNA-Seq-v2”. The immune scores and stroma scores here were calculated by ESTIMATE (Estimation of Stromal and Immune cells in Malignant Tumor tissues using Expression data) at backend of the tool (37). The file was downloaded as txt file, and stored locally as a raw file named “immune score_raw.csv”.

These four locally stored files were the inputs of the downstream analyses described in the Supplementary Data. The scores of CD4^+^ T cells, regulatory T cells (Tregs), M1 macrophages and M2 macrophages were estimated with R package *Immundeconv* (38). The CD8 T-cell–specific gene expression was estimated with the *Impute Cell Expression* function of *CIBERSORTx* running in group model, in which the cell-specific gene expression was impute with a built-in signature matrix file LM22 and merged into 10 major cell subsets including CD8 T cell (39, 40). The gene set enrichment analysis (GSEA) and result visualization were performed with GSEA 4.1.0 developed by UC San Diego and Broad Institute (41, 42). The other analyses and result visualizations were performed with the original codes executed with Python 3.8.5 on Anaconda 3 and R 4.0.2 on R studio. The original codes are available on GitHub (https://github.com/benchlover/SHP2_immunology).

### Incucyte-Based T-Cell Killing Assays

To induce antitumor immunity, 1  ×  10^5^ 4T07 cells were engrafted into the mammary fat pads of BALB/cJ mice via an intraductal injection (43). The enlarged spleens of tumor-bearing mice were harvested 3 weeks postinjection, and mechanically disrupted by grinding. CD8^+^ cells were isolated from the splenocytes using EasySep Mouse CD8^+^ T Cell Isolation Kit (STEMCELL Technologies Inc.) following the manufacturer's instructions. The D2.A1 cells were pretreated with growth factors and inhibitors listed in Supplementary Table S4 for 24 hours. The growth factors and inhibitors were washed-off before adding CD8^+^ cells. The ratio of tumor cells to T cells was 1:10. The coculture system was stained with Incucyte Cytotox Dye for Counting Dead Cells (Essen BioScience), and monitored using the Incucyte S3 (Essen BioScience).

### Flow Cytometry for MBC Cells *In Vitro*

The tumor cells were treated with growth factors and inhibitors listed in Supplementary Table S5 for 24 hours. The cells were harvested and stained with antibodies at 1:200 per antibody listed in Supplementary Table S6 for 45 minutes at 4°C in the dark. The stained cells were washed with PBS once and fixed by 10% formaldehyde. Flow cytometry was performed using Guava EasyCyte System (Millipore). The results were analyzed with FlowJo (7.6.1) software.

### Immunoblotting

Immunoblotting was performed as previously described (19). Briefly, the treated cells were harvested and lysed with modified RIPA lysis buffer (43). The concentration of lysates was determined by Pierce BCA Protein Assay Kit (Thermo Scientific). After SDS-PAGE and transfer, the polyvinylidene difluoride membranes (Millipore) were incubated with primary and secondary antibodies listed in Supplementary Table S7. Results were collected using the ChemiDoc Gel Imaging System (Bio-Rad) and LI-COR imaging (LI-COR Biosciences).

### RNA Isolation and Quantitative Real-time PCR Analysis

The process was performed as previously described (44). Briefly, total RNA from treated tumor cells was isolated with the EZNA total RNA kit (Omega BioTek). Then, the cDNA was synthesized with the Verso cDNA Synthesis Kit (Thermo Scientific) following the manufacturer's instructions. Quantitative real-time PCR systems were prepared with SYBR Green Master Mix (Thermo Scientific) and amplified with CFX Connect real-time PCR detection system (Bio-Rad). The primer set (forward 5′-CTCGCCTGCAGATAGTTCCC-3′, reverse 5′-GGGAATCTGCACTCCATCGT-3′) was used to detect mouse PD-L1. The primer set (forward 5′-CAACTTTGGCATTGTGGAAGGGCTC-3′, reverse 5′-GCAGGGATGATGTTCTGGGCAGC-3′) was used to detect mouse GAPDH. The results were normalized to GAPDH.

### Statistical Analysis

A Student *t* test was used for comparing differences between two groups of measurements in analyses of immune composition and *in vitro* assays. A Mann–Whitney *U* test was used for comparing differences between two patient groups with differential CD4^+^ T-cell, Tregs, M1 macrophages, and M2 macrophages’ infiltration; while a Student *t* test was used for the comparison in analyses of other clinical datasets. Group measurements of *in vivo* assays were compared with a Mann–Whitney nonparametric test. Error bars show the SEM. No exclusion criteria were used in these studies. All statistical tests were appropriate in which the groups are assumed with similar variance.

### Data Availability

The data analyzed in this study were obtained from Firebrowse at http://firebrowse.org/ and ESTIMATE online tool at https://bioinformatics.mdanderson.org/estimate/disease.html.

## Results

### Pharmacologic Inhibition of SHP2 Inhibits MBC

**FIGURE 1:**
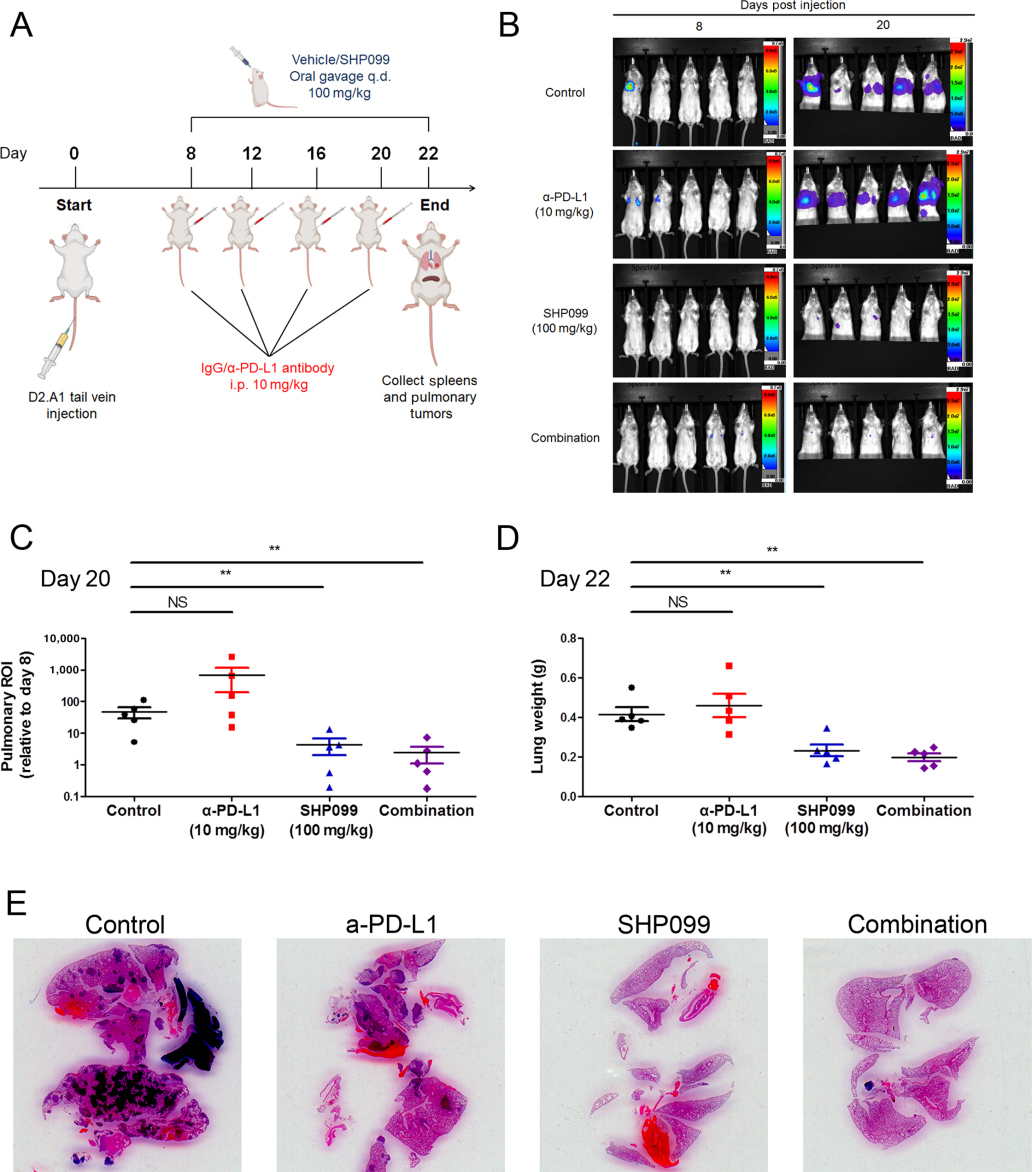
Pharmacologic inhibition of SHP2 inhibits MBC. **A,** Schematic of the study combining SHP099 with PD-L1 antibodies to treat mice bearing D2.A1 pulmonary tumors. Elements in the scheme were created using BioRender. **B,** Representative bioluminescent images of pulmonary D2.A1 growth at day 8 and day 20 postinjection. **C,** Bioluminescent values from pulmonary regions of interest (ROI) quantified as the ratio of day 20 to day 8 postinjection (**, *P* < 0.01, *n*  =  5 mice per group). **D,** Plots comparing the wet lung weights of the mice at day 22 postinjection (**, *P* < 0.01, *n*  =  5 mice per group). **E,** Representative H&E staining of lung histologic sections at day 22 postinjection.

We previously reported that depletion of fibroblast growth factor receptor 1 (FGFR1) increases infiltration of CD8^+^ lymphocytes into pulmonary tumors (43). SHP2 is a key node in FGFR1 and other RTK signaling. Therefore, we hypothesized that systemic SHP2 inhibition may also reprogram the TME of pulmonary metastases. To address this hypothesis, D2.A1 cells, a murine model of FGFR1-amplified MBC, were inoculated into mice via the lateral tail vein. Eight days after the tail vein injection, pulmonary tumor–bearing mice were treated with SHP099 and/or α-PD-L1 antibodies (Fig. 1A). As determined by bioluminescent imaging and wet pulmonary weights, the 12-day treatment course of α-PD-L1 antibody did not significantly inhibit the pulmonary tumor growth of D2.A1 cells. In contrast, the growth of D2.A1 tumors in the lungs was significantly reduced by SHP099 alone and when combined with α-PD-L1 antibodies (Fig. 1B–E; Supplementary Fig. S1A and S1B). We did not observe significant weight loss of the mice or a significant change in spleen weight with any of the therapies (Supplementary Fig. S1C and S1D). These data suggest that inhibition of SHP2 can effectively inhibit the pulmonary growth of a syngeneic MBC model that is resistant to ICB.

### Pharmacologic Inhibition of SHP2 Relieves T-Cell Exhaustion and Reprograms the Tumor–Immune Microenvironment

**FIGURE 2:**
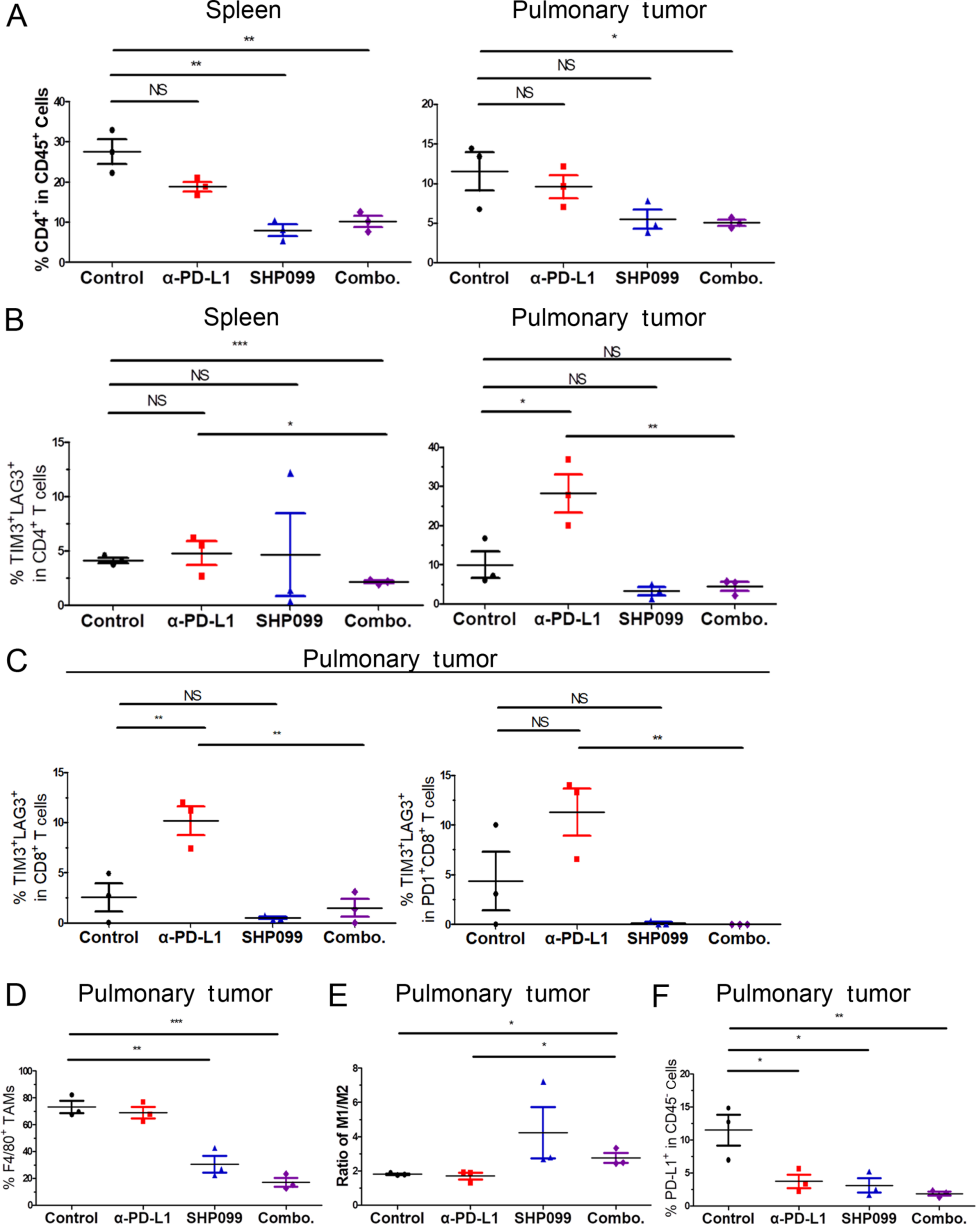
Pharmacologic inhibition of SHP2 relieves T-cell exhaustion and reprograms the tumor–immune microenvironment. **A,** Quantification of CD4^+^ population as a frequency of CD45^+^ cells in isolated spleens (left) and lung tissues (right) of each group. **B,** Quantification of TIM3^+^LAG3^+^ population as a frequency of CD45^+^CD4^+^ cells in isolated spleens (left) and lung tissues (right) of each group. **C,** Quantification of TIM3^+^LAG3^+^ population as a frequency of CD45^+^CD8^+^ cells (left) and CD45^+^CD8^+^PD-1^+^ cells (right) of in isolated lung tissues of each group. **D,** Quantification of F4/80^+^ population as a frequency of CD45^+^CD11b^+^ cells in isolated lung tissues of each group. **E,** Plots comparing the ratio of CD86^+^ and CD206^+^ in F4/80^+^ population as M1/M2 in isolated lung tissues of each group. **F,** Quantification of PD-L1^+^ population as a frequency of CD45^−^ cells in isolated lung tissues of each group. In all panels. *, *P* < 0.05; **, *P* < 0.01; ***, *P* < 0.001; *n* = 3.

To identify how the TME and peripheral immune composition are affected by systemic SHP2 inhibition, we collected the pulmonary tumors and spleens of pulmonary tumor–bearing mice after 14 days of treatment. As shown in Fig. 1E, pulmonary metastasis was reduced with SHP099 treatment to such an extent that precluded accurate IHC analyses. Therefore, tissues were dissociated to single cells and analyzed by flow cytometry with desired gating strategies (Supplementary Fig. S2). Flow cytometry revealed that the percentage of CD4^+^ cells within the CD45^+^ splenic population significantly decreased upon combination of SHP099 and α-PD-L1 (Fig. 2A, left; Supplementary Fig. S3A). In contrast, the percentage of CD8^+^ cells increased (Supplementary Fig. S3B). Similar results in the percentage of CD4^+^ population were observed in the tumor-infiltrating lymphocytes from pulmonary tumors, but there was no difference in the percentage of CD8^+^ population (Fig. 2A, right; Supplementary Fig. S3C and S3D). To further investigate the status of these T cells, we focused on the exhaustion markers, lymphocyte activating protein 3 (LAG3) and T-cell immunoglobulin domain and mucin domain 3 (TIM3). The percentage of TIM3^+^LAG3^+^ in CD4^+^ T cells was increased by α-PD-L1, and this exhaustion was significantly abolished in the spleen and pulmonary tumor when SHP099 was added in the combination (Fig. 2B; Supplementary Fig. S4A). In spleens, the percentage of TIM3^+^LAG3^+^ in CD4^+^ T cells was also significantly reduced with combination of SHP099 and α-PD-L1 antibody compared with the control group (Fig. 2B, left; Supplementary Fig. S4A, left). Similar results were also observed in the percentage of TIM3^+^ in CD4^+^ T cells in spleens and pulmonary tumors (Supplementary Fig. S4B and S4C). Similarly, the percentage of exhausted CD8^+^ T cells defined as TIM3^+^LAG3^+^ in pulmonary tumors was significantly increased by α-PD-L1 antibody, which was significantly abolished by the addition of SHP099 (Fig. 2C; Supplementary Fig. S4D and S4E). The results were confirmed with the percentage of LAG3^+^, TIM3^+^, and PD-1^+^ cells in CD8^+^ T cells from pulmonary tumors (Supplementary Fig. S4F and S4G). Taken together, these data suggest that SHP099 not only adjusts T-cell composition but also relieves the T-cell exhaustion induced by ICB.

Next, we focused on characterization of myeloid cells from the pulmonary tumors to decipher the impact SHP099 has on tumor associated macrophages (TAMs) (10). The percentage of CD11b^+^ monocytes was induced by α-PD-L1, which was significantly reduced by addition of SHP099 (Supplementary Fig. S5A). The percentage of TAMs (F4/80^+^ in CD11b^+^ monocytes) was significantly reduced with SHP099 and the combination therapy as compared with the control (Fig. 2D; Supplementary Fig. S5B). The percentage of M1-polarized macrophages (CD86^+^ in F4/80^+^CD11b^+^CD45^+^ cells) was significantly reduced by α-PD-L1 antibody, which was rescued by SHP099; while the percentage of M2-polarized macrophages (CD206^+^ in F4/80^+^CD11b^+^CD45^+^ cells) was significantly reduced by SHP099 and combination therapy (Supplementary Fig. S5C and S5D). Hence, the ratio of M1/M2 macrophages increased with SHP099 and combination therapy (Fig. 2E). These data demonstrated that systemic SHP2 inhibition reduced total TAMs and shifted the remaining population toward the tumor-suppressive M1 phenotype. To confirm that the α-PD-L1 antibody was on-target, we focused on the PD-L1 levels in the tumor cells. The percentage of PD-L1^+^ cells in CD45^−^ population was significantly reduced by all the treatments, and the reduction was enhanced with combination therapy (Fig. 2F; Supplementary Fig. S5E).

### Depletion of Tumor Cell–Autonomous SHP2 Reduces Pulmonary Metastasis, Alters Immune Profiles, and Prevents T-Cell Exhaustion

**FIGURE 3:**
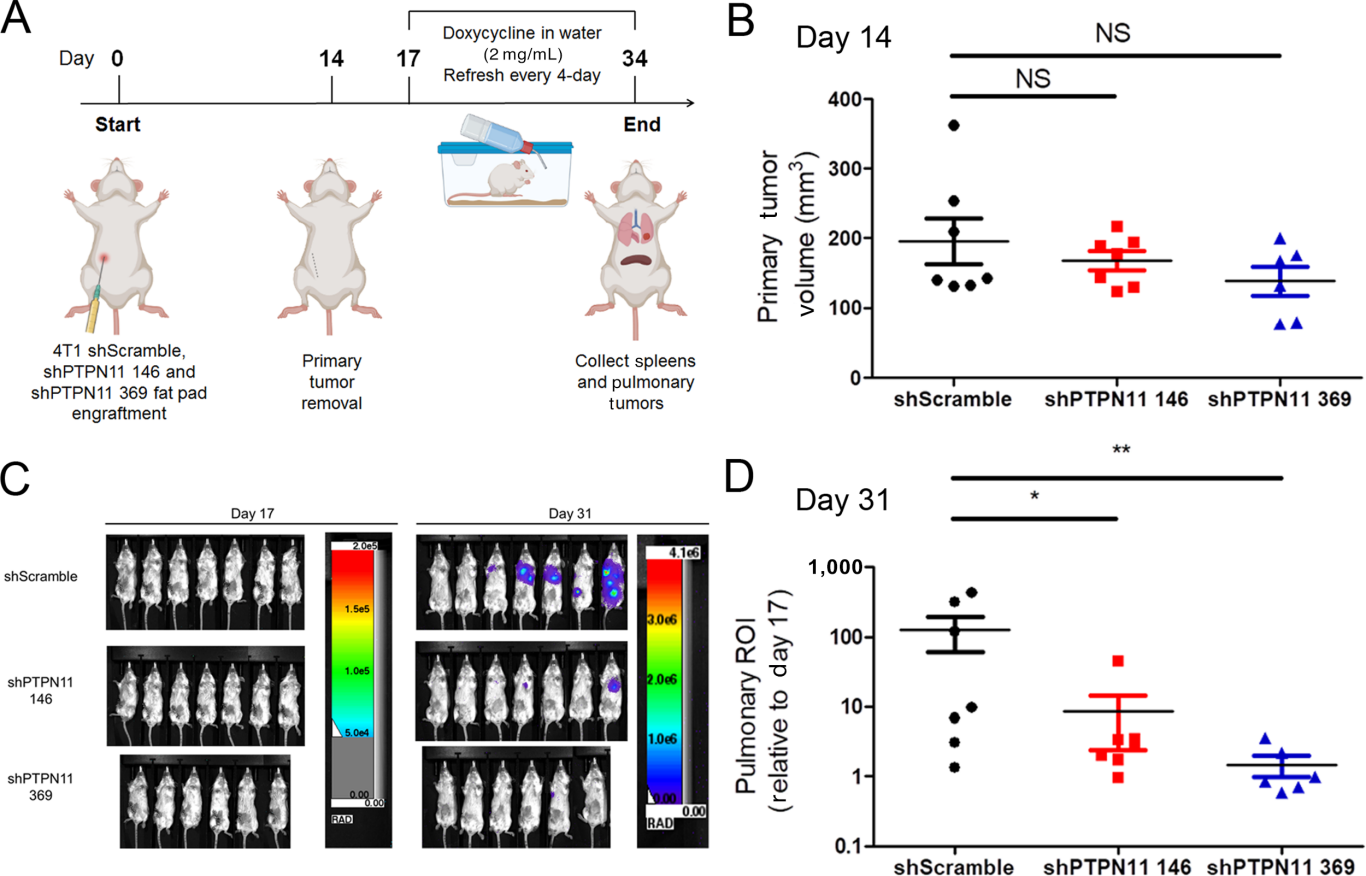
Depletion of tumor cell–autonomous SHP2 reduces pulmonary metastasis, alters immune profiles, and prevents T-cell exhaustion. **A,** Schematic of the study using doxycycline-inducible depletion of SHP2 in 4T1 cells. Elements in the scheme were created using BioRender. BALB/c mice (*n* = 7 mice per group for shScramble and shPTPN11 146, *n* = 6 mice per group for shPTPN11 369) were orthotopically engrafted with 4T1 cells (5 × 10^4^) via intraductal injection. Primary tumors were surgically removed 2 weeks following the injection. Doxycycline was administrated in drinking water at 2 mg/mL 3 days following the removal of primary tumors. **B,** Plots comparing the primary tumor volume at day 14 postinjection. NS, no significance. **C,** Representative bioluminescent images of 4T1 pulmonary metastasis at day 17 and day 31 postinjection. **D,** Bioluminescent values from pulmonary ROI quantified as the ratio of day 31 to day 17 postinjection (*, *P* < 0.05; **, *P* < 0.01).

We next sought to evaluate the specific contribution of tumor cell–autonomous SHP2 to MBC pulmonary metastasis and immune composition. To this end, we utilized doxycycline-inducible depletion of SHP2 in the 4T1 orthotopic model of MBC (19). This model of spontaneous metastasis nicely recapitulates MBC disease progression as primary tumors are grown, removed, and tracked for metastasis using bioluminescence (45). Using doxycycline inducible depletion, we were able to specifically deplete SHP2 in disseminated tumor cells only after removal of the primary tumor (Fig. 3A; ref. 46). As expected, we did not observe changes in primary tumor growth (Fig. 3B). In contrast, the 14-day administration of doxycycline to induce SHP2 depletion significantly reduced pulmonary metastases as determined by bioluminescent imaging (Fig. 3C and D; Supplementary Fig. S6A). No significant weight loss was observed with doxycycline (Supplementary Fig. S6B). The efficiency of doxycycline administration to induce shRNA expression was verified by measuring GFP signal from the lungs as eGFP and shRNA are under the control of the same tet-responsive element. The GFP signal observed was consistent with the differential efficiency of the shRNA constructs targeting PTPN11 (Supplementary Fig. S6C). The reduction of pulmonary metastases was confirmed by decreases in pulmonary wet weights and *ex vivo* bioluminescent imaging of the lungs upon necropsy (Supplementary Fig. S6D and S6E).

**FIGURE 4:**
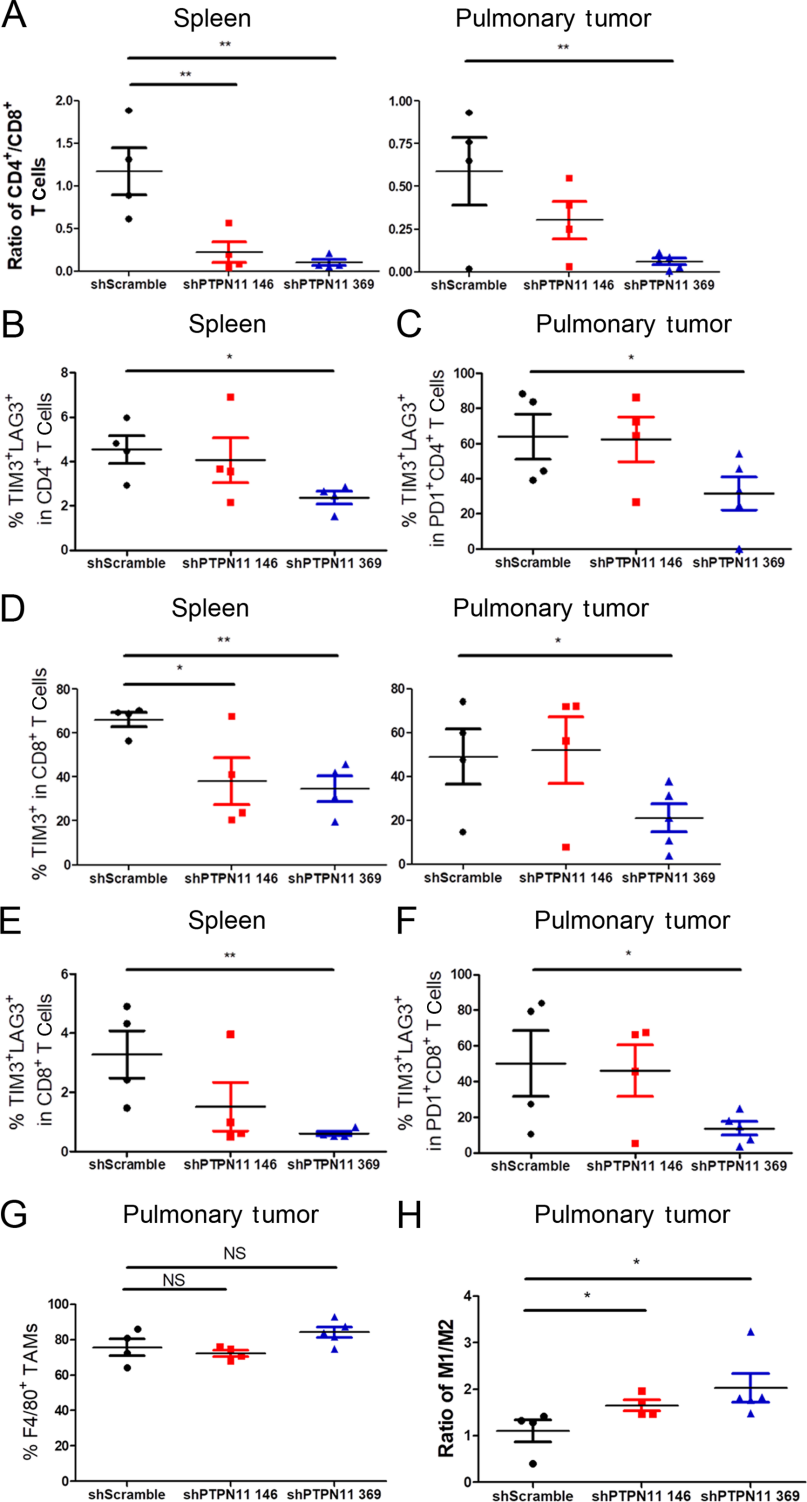
Depletion of tumor cell–autonomous SHP2 relieves immune suppression. **A,** Plots comparing the ratio of the frequency of CD4^+^ and CD8^+^ as CD4/CD8 in isolated spleen (left) and lung tissues (right) of each group. **B,** Quantification of TIM3^+^LAG3^+^ population as a frequency of CD45^+^CD4^+^ cells in isolated spleens of each group. **C,** Quantification of TIM3^+^LAG3^+^ population as a frequency of CD45^+^CD4^+^PD-1^+^ cells in isolated lung tissues of each group. **D,** Quantification of TIM3^+^ population as a frequency of CD45^+^CD8^+^ cells in isolated spleens (left) and lung tissues (right) of each group. **E,** Quantification of TIM3^+^LAG3^+^ population as a frequency of CD45^+^CD8^+^ cells in isolated spleens of each group. **F,** Quantification of TIM3^+^LAG3^+^ population as a frequency of CD45^+^CD8^+^PD-1^+^ cells in isolated lung tissues of each group. **G,** Quantification of F4/80^+^ population as a frequency of CD45^+^CD11b^+^ cells in isolated lung tissues of each group. **H,** Plots comparing the ratio of CD86^+^ and CD206^+^ in F4/80^+^ population as M1/M2 in isolated lung tissues of each group. In all panels. NS, no significance. *, *P* < 0.05; **, *P* < 0.01; ***, *P* < 0.001; *n* = 4 for shScramble and shPTPN11 146, *n* = 5 for shPTPN11 369 in pulmonary tumor panels, *n* = 4 for each group in spleen panels.

To elucidate changes in the TME following tumor cell–specific depletion of SHP2, the pulmonary tumors and spleens of the mice were collected after 17 days of doxycycline administration. Cells were analyzed by flow cytometry using the desired gating strategies (Supplementary Fig. S7). Similar to our approach in Fig. 2, we characterized T-cell composition, T-cell exhaustion, and TAM composition. Upon SHP2 depletion, the percentage of CD4^+^ cells within the CD45^+^ population of the spleen significantly decreased and the percentage of CD8^+^ cells significantly increased, which was observed in pulmonary tumors as well (Supplementary Fig. S8). Hence, the ratio of CD4^+^/CD8^+^ T cells decreased significantly with depletion of SHP2 (Fig. 4A). The percentage of exhausted CD4^+^ T cells, described as TIM3^+^LAG3^+^, was reduced in spleens and pulmonary tumors by depletion of tumor cell–autonomous SHP2 (Fig. 4B and C; Supplementary Fig. S9A and S9B). The results were confirmed with the percentage of LAG3^+^ and TIM3^+^ in CD4^+^ T cells from spleens (Supplementary Fig. S9C and S9D). In addition, the percentage of TIM3^+^ in CD8^+^ T cells was significantly reduced by SHP2 depletion in spleen and pulmonary tumor (Fig. 4D; Supplementary Fig. S10A). The percentage of exhausted CD8^+^ T cells described as TIM3^+^LAG3^+^ was also reduced with SHP2 depletion (Fig. 4E and F; Supplementary Fig. S10B and S10D). The reduction of exhausted CD8^+^ T cells described as LAG3^+^ was also observed in spleen (Supplementary Fig. S10E). We next focused on TAM composition. There was reduction of CD11b^+^ monocytes, but no change in the percentage of F4/80^+^ TAMs with tumor cell–autonomous SHP2 depletion (Fig. 4G; Supplementary Fig. S11A and S11B). The percentage of M1-polarized macrophages increased, and the percentage of M2-polarized macrophages decreased, which led to significant elevation of M1/M2 ratio upon SHP2 depletion (Fig. 4H; Supplementary Fig. S11C and S11D).

Taken together, these data suggest that, similar to systemic inhibition of SHP2, targeted depletion of the SHP2 in pulmonary metastases leads to alterations of both the peripheral and tumor-infiltrating immune components.

### Phosphorylation of SHP2 Predicts Immune Profiles in Patients with MBC

**FIGURE 5:**
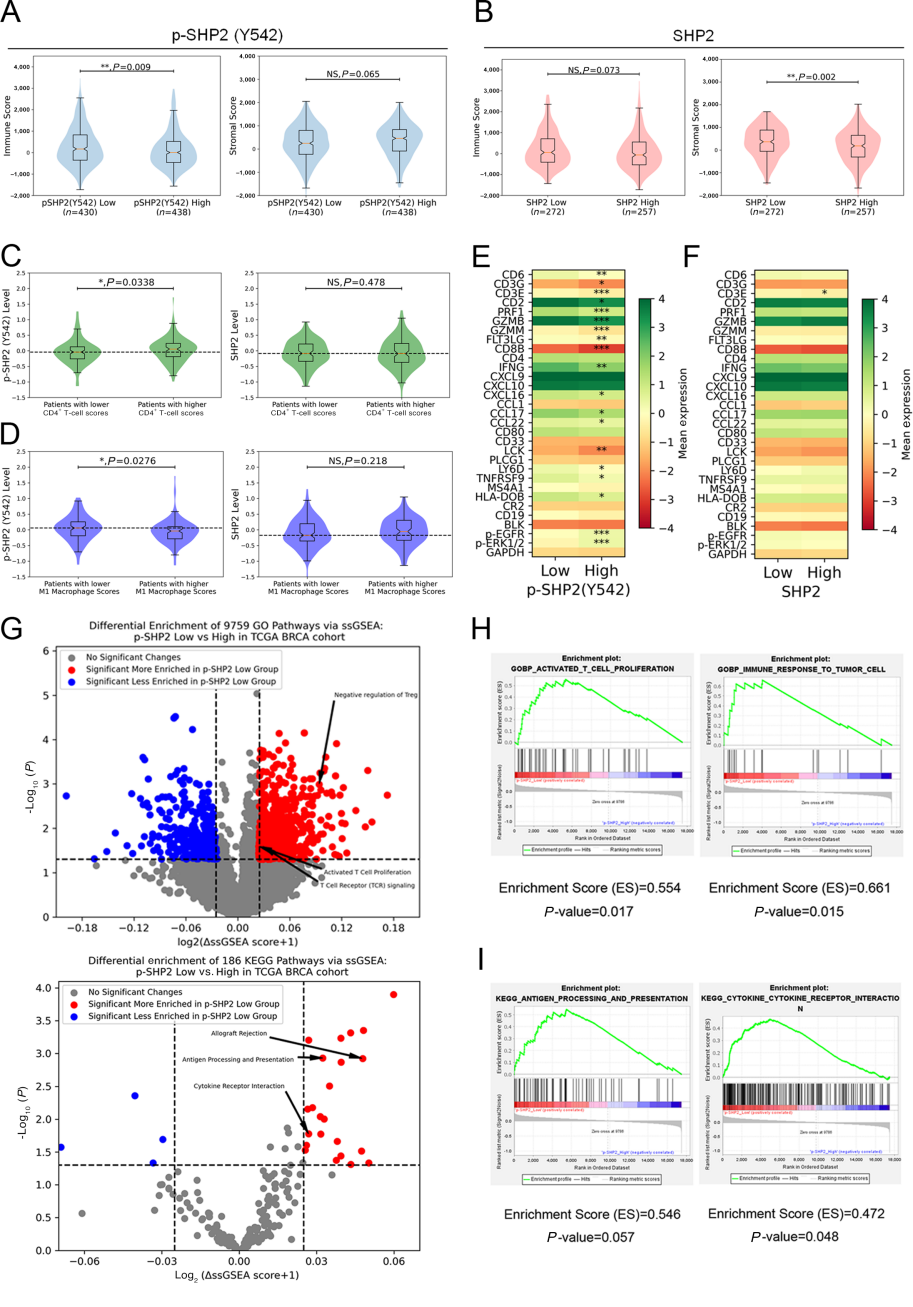
Phosphorylation of SHP2 predicts immune profiles in patients with MBC. **A, B,** Violin and box plots comparing the differential immune scores and stroma scores in patients grouped by phosphorylation levels of SHP2 at Y542 (**A**) or expression levels of SHP2 (**B**). **C, D,** Violin and box plots comparing the differential phosphorylation levels of SHP2 at Y542 and expression levels of SHP2 in patients grouped by CD4^+^ T-cell infiltration (**C**) and M1 Macrophage infiltration (**D**) levels. Heatmaps comparing the differential gene expression in patients grouped by phosphorylation levels of SHP2 at Y542 (**E**) or expression levels of SHP2 (**F**). *, *P* < 0.05; **, *P* < 0.01; ***, *P* < 0.001. **G,** Volcano plots demonstrating pathways of differential ssGSEA scores in patients grouped by phosphorylation levels of SHP2 at Y542 with statistical significance. Specific pathways of interest are annotated. GSEA plots, Enrichment scores and *P* values of the key pathways from GO (**H**) and KEGG (**I**) enriched in patients with lower phosphorylation levels of SHP2 at Y542.

To find clinical evidence to correlate SHP2 with immune profiles in MBC patients, we analyzed the BRCA cohort of TCGA datasets. We have previously demonstrated that phosphorylation of SHP2 at Y542 is associated with decreased patient survival in this cohort (19). Here we found that patients with higher phosphorylation of SHP2 at Y542 had significant lower immune scores, indicating reduced immune cell infiltration in tumors (Fig. 5A). In contrast to Y542 phosphorylation, differential expression of total levels of SHP2 was not predictive of immune scores, but did correlate with a reduced stromal score in these patients (Fig. 5B). Consistent with our animal data, patients with higher phosphorylation levels of SHP2 had higher CD4^+^ T-cell and lower M1 Macrophages infiltration predicted by *Immundeconv* (Fig. 5C and D). M2 macrophage infiltration also increased in patients with higher phosphorylation levels of SHP2, while no difference was observed in Treg infiltration (Supplementary Fig. S12A and S12B). Next, we examined differential expression of specific immune-related markers that correlated with differential phosphorylation of SHP2. We found that the key genes in T-cell composition, T-cell activation and antigen presentation, including *PRF1, CD8B, GZMB, LCK, IFNG,* and *HLA-DOB*, were significantly associated with the phosphorylation of SHP2 at Y542, but not total expression levels of SHP2 (Fig. 5E and F). As immune-related markers might not be exclusively expressed in T cells, we imputed the CD8 T cell–specific gene expression and further confirmed elevated levels of T-cell exhaustion markers, TIM3 (*HAVCR2*) and PD-1 (*CD247*), in patients with higher phosphorylation levels of SHP2 (Supplementary Fig. S12C). Using single-sample GSEA (ssGSEA), we examined enriched KEGG and GO pathways in patients with differential levels of SHP2 phosphorylation. Using this approach, we found that pathways of activated T-cell proliferation and antigen processing & presentation were significantly enriched in patients with lower phosphorylation of SHP2 (Fig. 5G). The enrichment of these two pathways was confirmed with GSEA (Fig. 5H and I). These results further strengthen the notion that SHP2 activation via phosphorylation at Y542 contributes the weaker immune profiles in patients with MBC.

### SHP2 Facilitates Growth Factor–Induced Resistance to T-Cell Cytotoxicity via Regulation of PD-L1

**FIGURE 6:**
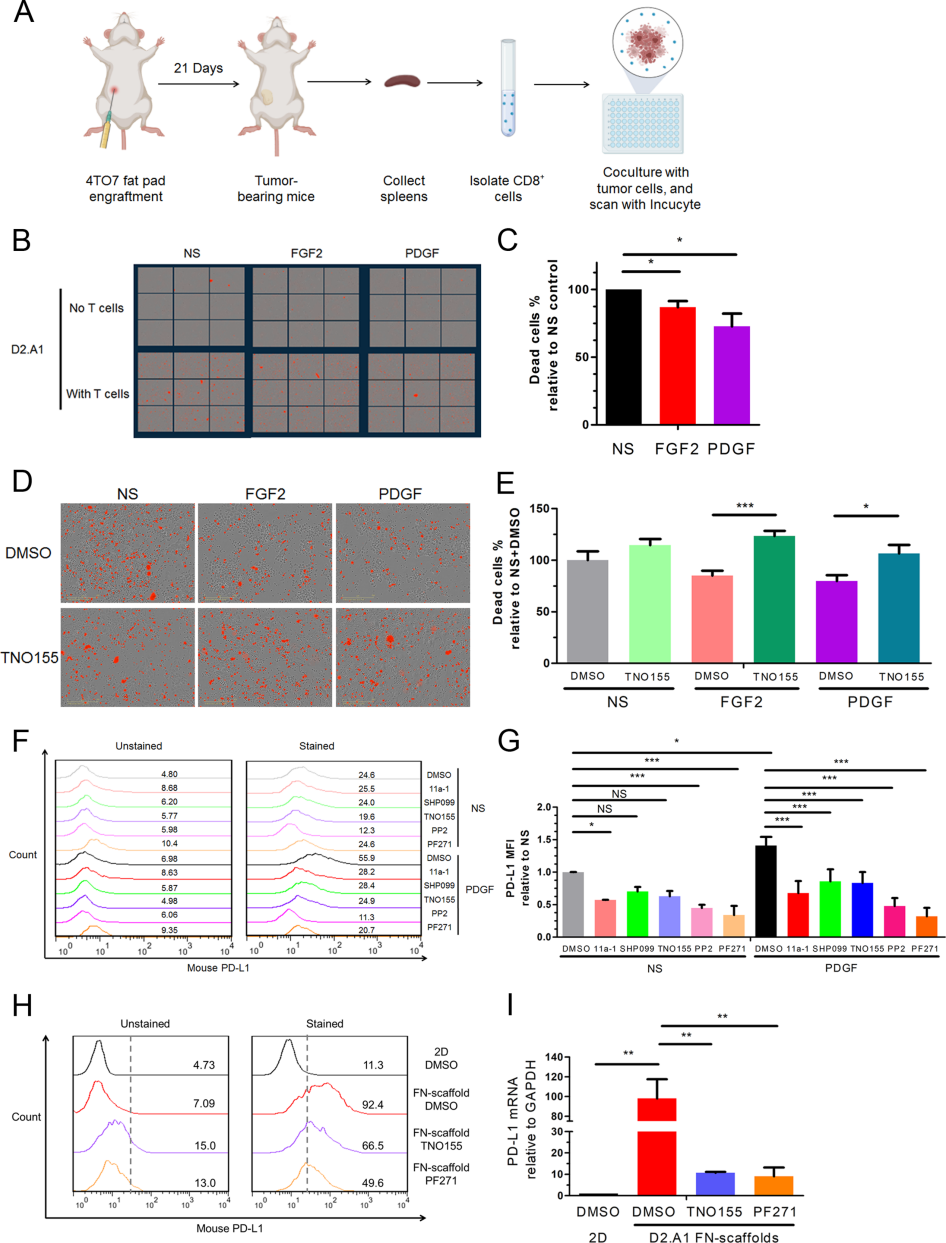
SHP2 facilitates growth factor–induced resistance to T-cell cytotoxicity via regulation of PD-L1. **A,** Schematic of T-cell cytotoxicity assays. Elements in the scheme were created using BioRender. CD8^+^ T cells are isolated from the spleens of tumor-bearing mice. The MBC cells are treated with growth factors and inhibitors, and cocultured with CD8^+^ T cells. The dead cells are quantified by with Incucyte imaging. **B,** Representative images of the MBC cells treated with FGF2 (20 ng/mL) and PDGF (100 ng/mL) at 1 hour following coculturing with T cells. The MBC cells without T cells served as background. **C,** Bar graph comparing the percentage of dead cell counts of FGF2 and PDGF groups to the no stimulation (NS) group. *, *P* < 0.05; *n* = 4 individual repeats. **D,** Representative images of the MBC cells treated with FGF2/PDGF and TNO155 (5 μmol/L) at 1 hour following coculture with T cells. **E,** Bar graph comparing the percentage of dead cell counts with different treatments. *, *P* < 0.05; ***, *P* < 0.001, *n* = 9. Histogram of cell surface PD-L1 using flow cytometry (**F**) and bar graph (**G**) comparing fold changes of PD-L1 MFIs in D2.A1 cells induced by PDGF and treated with different inhibitors. *, *P* < 0.05; **, *P* < 0.01; ***, *P* < 0.001; *n* = 3. **H,** Histogram of cell surface PD-L1 using flow cytometry in D2.A1 cells induced by 3D culture on a fibronectin-coated scaffold and treated with the indicated inhibitors. **I,** Bar graph comparing fold change of PD-L1 mRNA of D2.A1 cells cultured and treated as in **H**. **, *P* < 0.01; *n* = 3.

To investigate how SHP2 signaling in tumor cells contributes to T-cell exhaustion, we utilized a T-cell cytotoxicity assay. In this assay, CD8^+^ T cells were isolated from the spleens of tumor-bearing mice and cocultured with MBC cells (Fig. 6A). After coculture with T cells, we could readily observe tumor cell cytotoxicity, a result that could be enhanced by addition of SHP099 and TNO155 treatments (Supplementary Fig. S13A and S13B). Given that RTK signaling is one of the signaling inputs that is dependent on SHP2, we treated the D2.A1 cells with FGF2 or PDGF before coculturing with T cells (19). Pretreatment with these growth factors significantly reduced T cell–mediated cytotoxicity (Fig. 6B and C). To verify the role of tumor cell–autonomous SHP2 in this immune protection, D2.A1 cells were treated with these two growth factors and TNO155. TNO155 rescued T-cell cytotoxicity in both cases (Fig. 6D and E). The ability of SHP2 inhibition in tumor cells to prevent the ability of growth factors to protect tumor cells from T cell–mediated killing was confirmed by doxycycline-inducible depletion of SHP2 (Supplementary Fig. S13C and S13D). Moreover, similar results were achieved with an α-PD-L1 antibody (Supplementary Fig. S13E and S13F). As shown in Fig. 2F, PD-L1 in tumor cells was also significantly reduced with systemic SHP2 inhibition *in vivo*. We further hypothesized that the SHP2 might regulate PD-L1 expression levels downstream of RTK signaling. Flow cytometry revealed that FGF2 and PDGF significantly induced PD-L1 levels in D2.A1 cells (Supplementary Fig. S14A and S14B). Quantitative PCR demonstrated that the induction of PD-L1 by growth factors was regulated transcriptionally (Supplementary Fig. S14C). Treatments of 11a-1, SHP099, TNO155, PP2 (Src inhibitor), and trametinib (MEK inhibitor) abolished the induction of PD-L1 by PDGF (Fig. 6F and G; Supplementary Fig. S15A and S15B). In BT549 cells, PD-L1 could be significantly induced by FGF2 and EGF (Supplementary Fig. S15C and S15D). The ability of SHP2 inhibition to reduce growth factor–induced PD-L1 was also confirmed in BT549 cells with treatments of TNO155 (Supplementary Fig. S15E and S15F). In addition to RTK signaling, ECM signaling is another signaling input of SHP2 and phosphorylation of SHP2 at Y542 is elevated in MBC cells under 3D culture environment with fibronectin-coated tessellated scaffolds (19, 47). Flow cytometry demonstrated that PD-L1 in D2.A1 cells was significantly elevated when cultured on fibronectin-coated scaffolds compared with tissue culture polystyrene (2D culture; Supplementary Fig. S15G). Similar results were observed with laminin-coated scaffolds and other MBC cell lines (Supplementary Fig. S15G). The elevation of PD-L1 with fibronectin-coated scaffolds could be significantly abolished by TNO155 and PF271 (FAK inhibitor) transcriptionally, but not trametinib (Fig. 6H and I; Supplementary Fig. S15H). The involvement of SHP2 in the regulation of PD-L1 was confirmed with MBC cells with doxycycline-inducible depletion of SHP2 (Supplementary Fig. S15I). These findings indicate that upregulation of PD-L1 expression by both growth factor–mediated RTKs signaling and 3D culture environment with ECM signaling in MBC cells is mediated through SHP2.

### SHP2 Regulates the Expression of MHC Class via a Balance Between MAPK and STAT1 Signaling in MBC Cells

**FIGURE 7:**
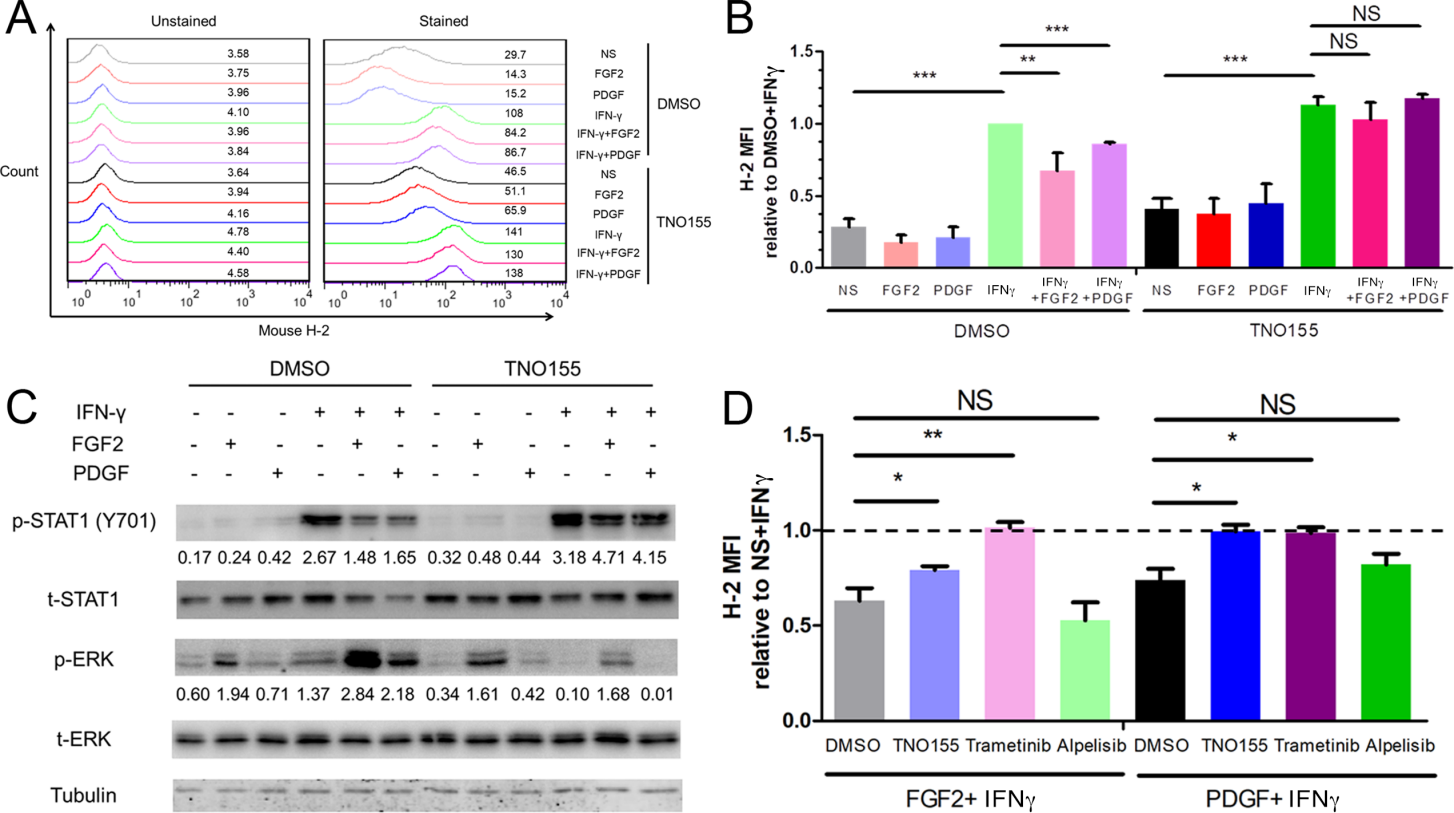
SHP2 regulates the expression of MHC class I via a balance between MAPK and STAT1 signaling in MBC cells. **A,** Histogram of cell surface analysis of H-2 in D2.A1 cells treated with different growth factors, mouse IFNγ (200 ng/mL) and TNO155 (5 μmol/L). **B,** Bar graph comparing fold change of H-2 MFIs induced by different growth factors, IFNγ and TNO155 compared with DMSO+ IFNγ. NS, not significant; **, *P* < 0.01; ***, *P* < 0.001; *n* = 3. **C,** Immunoblotting showing differential STAT1 and ERK1/2 phosphorylation in D2.A1 cells treated with different growth factors, IFNγ, and TNO155. **D,** Bar graph comparing fold change of H-2 MFI induced by different growth factors and IFNγ compared with IFNγ alone with different inhibitors in D2.A1 cells. NS, not significant; **, *P* < 0.01; ***, *P* < 0.001; *n* = 3.

Besides the activated T-cell proliferation, we found that antigen processing and presentation was significantly more enriched in patients with lower phosphorylation of SHP2 (Fig. 5G–I). Moreover, we found IFNG was significantly reduced in the patients with higher levels of SHP2 phosphorylation (Fig. 5E). Hence, we focused on the ability of SHP2 to regulate expression of MHC class I, which is critical for antigen presentation and mediated by IFN-γ (48, 49). Flow cytometry demonstrated that FGF2 and PDGF significantly limited that ability of IFNγ to induce expression of MHC class I (Fig 7A and B). Importantly, this effect was prevented upon treatment with TNO155 (Fig. 7A and B). Similar results were observed in BT549 cells with FGF2, EGF, and TNO155 (Supplementary Fig. S16A and S16B). Immunoblotting showed that TNO155 prevented phosphorylation of ERK1/2 induced by FGF2 and PDGF, and augmented the ability of IFNγ to induce STAT1 phosphorylation (Fig. 7C). Similarly, trametinib, but not alpelisib (PI3K inhibitor), also rescued IFNγ induced MHC class I in the presence of FGF2 and PDGF (Fig. 7D). Although PD-L1 is also regulated by IFNγ, the levels of PD-L1 were not significantly influenced by TNO155 or alpelisib, and even significantly induced by trametinib, under growth factors plus IFNγ (Supplementary Fig. S16C). The ability of SHP2 inhibition to rescue MHC class I expression under growth factor–stimulated conditions was further demonstrated by TNO155 enhancing IFNγ-induced T-cell cytotoxicity under PDGF-stimulated conditions (Supplementary Fig. S16D and S16E). These data suggest that SHP2 acts as a key node that regulates the balance between MAPK and STAT1 signaling, the targeting of which is capable of enhancing antigen presentation and increasing antitumor immunity.

## Discussion

Therapeutic benefits of ICB are limited for metastatic breast cancer, but novel targeted therapies to combine with ICB and enhance efficacy are emerging. Beyond tumor cells, the TME is a dynamic community composed of immune cells with diverse functions in response to a variety of stimuli. In the presence of ICBs, these complicated signaling pathways are engaged both in the tumor cells and immune cells to shift the balance between immunogenicity and immunosuppression in the TME. Recent findings have started to illustrate the potential benefits of combining SHP2 inhibitors with ICB, but the mechanisms by which targeting SHP2 enhances the effects of ICB, especially in a tumor cell autonomous manner, are yet to be fully elucidated (32, 50, 51). Herein, we demonstrate that tumor cell–autonomous SHP2 facilitates MBC metastasis and resistance to ICB via creating an immunosuppressive TME. Our working model is supported by separate studies illustrating that systemic targeting of SHP2 promotes antitumor immunity via mechanisms that go beyond direct effects on immune cells (52–55).

Our current study did not elucidate a combinatorial effect in terms of tumor growth between SHP099 and α-PD-L1, which might require further dosage optimization and timing, but we did observe the combination group achieved faster regression in pulmonary tumor burden, which could be a benefit from combination therapy (50, 51, 54). This adjuvant treatment approach allowed us to evaluate the role of SHP2 specifically in the progression of established metastatic tumors, as we initiated SHP2 inhibitor treatments or doxycycline induced depletion of SHP2 only after tail vein injection or primary tumor removal and once metastases were seeded. In addition, several studies indicate that LAG3 and TIM3 are key T-cell exhaustion markers, which contribute to the lack of an antitumor immune response upon ICB therapy (56–62). Consistent with these reports, α-PD-L1 antibody treatment alone did not significantly reduce the pulmonary growth of the syngeneic D2.A1 model of MBC, and resulted in high level expression of TIM3 and LAG3 in T-cells from tumor-bearing mice. Importantly, we demonstrate that T-cell exhaustion was abolished upon treatment with SHP099. The pattern of T-cell exhaustion in the TME upon α-PD-L1 antibody treatments is also supported by previous reports. For instance, both LAG3 and TIM3 were significantly upregulated upon α-PD-L1 antibody treatments in T cells from pulmonary tumors. In contrast, α-PD-L1 induction of these exhaustion markers did not elevate in CD4^+^ T cells from spleens, demonstrating that reprogramming tumor-infiltrating lymphocytes (TIL) is the key to improved therapeutic outcomes.

Besides T-cell exhaustion, the amount of T-cell infiltration and composition of TAMs within tumors are critical factors for the response to ICB (63–65). We observed that M1-polarized macrophages were significantly reduced by α-PD-L1. Our observation that SHP099 reduced TAMs and increased the M1/M2 ratio is supported by recent studies and suggests modulation of this myeloid compartment as a major contributing factor to the efficacy of SHP2-targeted therapies (32, 54, 66). Finally, we observed that SHP099 resulted in reduced numbers of CD4^+^ T cells in pulmonary tumors, but no increase in CD8^+^ infiltration was observed. In contrast, combination of SHP099 and α-PD-L1 led to a dramatic increase in splenic CD8^+^ T cells. Overall, these data suggest that increasing cytotoxic T-cell infiltration into metastatic tumors remains a challenge for immune therapy that is not overcome by SHP2 targeting.

As SHP2 is expressed in both tumor cells and immune cells, we sought to investigate how tumor cell–autonomous SHP2 contributes to immune escape by MBC. Indeed, previous reports suggest SHP2 in T cells is dispensable for their function and that the tumor-facilitating role of SHP2 lies in myeloid cells and tumor -associated endothelial cells (25, 26, 67). Using the doxycycline-inducible system we previously established, we demonstrate that depletion of SHP2 specifically in MBC cells reduces pulmonary metastasis (19, 46, 68). With depletion of SHP2 in tumor cells, reduction of CD4^+^ and induction of CD8^+^ T cells were observed in both pulmonary tumors and spleens, and the T-cell exhaustion markers were also reduced, matching the effects of systemic SHP2 inhibition. The enhancement of T-cell cytotoxicity by SHP2 inhibition in MBC cells was also confirmed with *in vitro* T-cell cytotoxicity assays. The M1/M2 ratio of TAMs was also modulated in similar fashion as compared with systemic inhibitors, but total TAM populations were not significantly changed. These findings are consistent with the notion that SHP2 function in tumor cells can influence the lymphoid, but not the myeloid components of the metastatic TME (69). Overall, our doxycycline-inducible depletion of SHP2 in MBC cells, allowed the first investigation into role of tumor cell–autonomous SHP2 specifically within the metastatic TME.

The clinical significance of phosphorylation of SHP2 has been supported by multiple studies, including those herein, where we demonstrate that phosphorylation of SHP2 at Y542 is a promising marker to predict immune profiles in patients with MBC (19, 70, 71). These findings further solidify the correlation between phosphorylation of SHP2 and immune response in patients with MBC. We did observe changes in CD4 T-cell infiltration upon SHP2 targeting. A relationship between SHP2 phosphorylation and Treg was not supported by clinical datasets. However, further characterization of CD4 phenotypes in tumor-bearing and tumor-naïve mice could yield insight into the impact of SHP2 inhibition on immune exhaustion. Mechanistically, we demonstrated that tumor cell–autonomous SHP2 regulates CD8^+^ T-cell cytotoxicity downstream of multiple growth factors via regulation of PD-L1 and MHC class I. Our studies also identify the ability of 3D culture environment with ECM signaling to promote PD-L1 via SHP2. Studies to determining the mechanistic details behind this event are ongoing, but our studies herein strongly suggest that the ability of SHP2 to balance STAT1 and MAPK signaling always for its regulation of PD-L1 expression at several levels (72).

**FIGURE 8:**
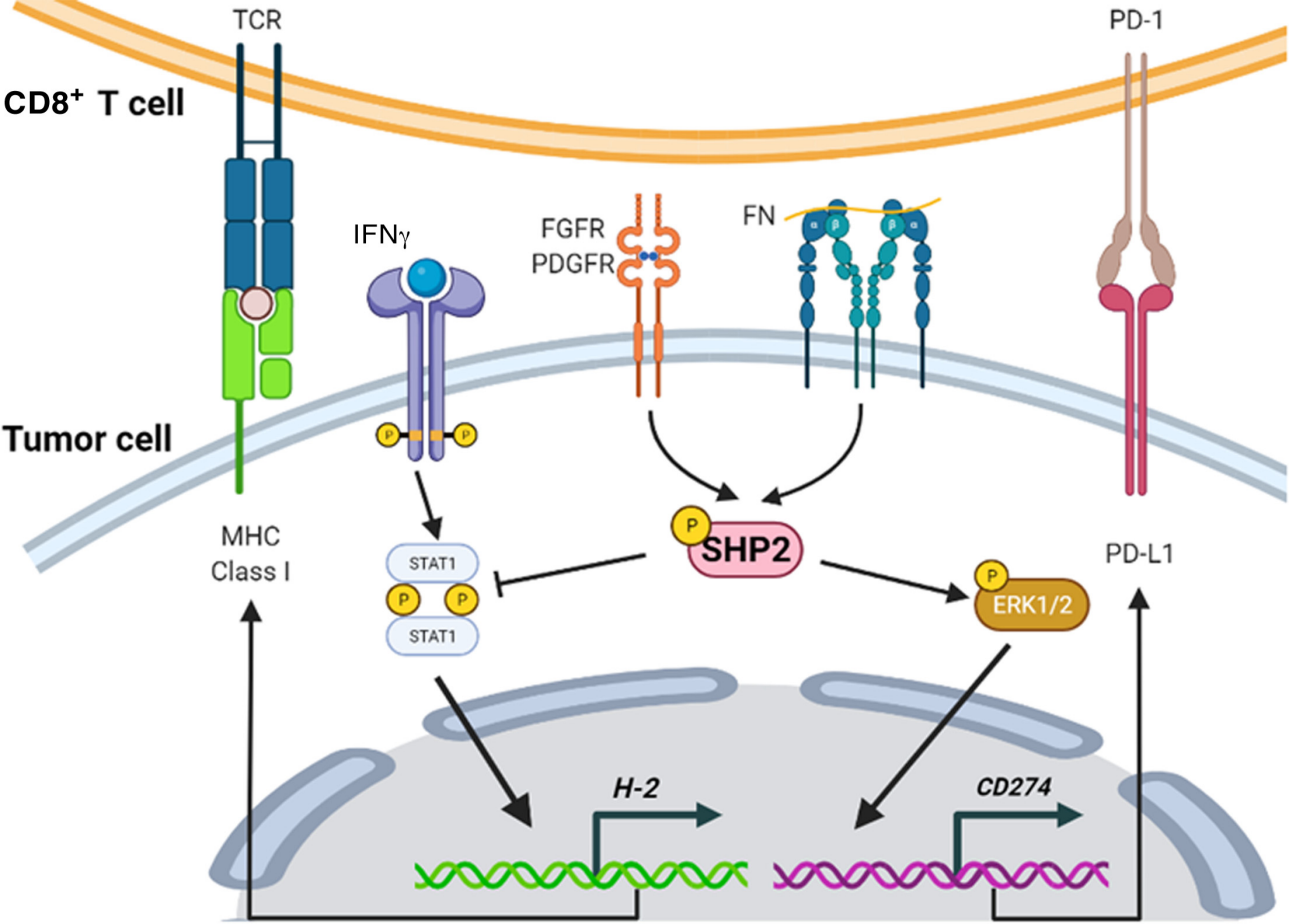
Tumor cell–autonomous SHP2 is a key signaling node in response to dynamic TME to induce immune suppression via regulating PD-L1 and MHC class I. SHP2 contributes to various downstream signaling pathways including PD-L1 and MHC class I to facilitate immune suppression in response to a varieties of additional signaling inputs in TME, such as growth factor receptor signaling. Figure was created using BioRender.

In summary, we show that tumor cell–autonomous SHP2 is a key signaling node by which MBC cells induce immune suppression from a variety of signaling inputs within the TME (Fig. 8). We establish phosphorylation of SHP2 at Y542 as a predictive marker of immune profiling in patients with MBC. Our studies also provide further mechanistic insights into clinical approaches pursuing combination strategies using SHP2 inhibition with ICB (NCT04000529).

## Supplementary Material

Supplementary Information SI1 This is the code information for publicly available data analysis.

Supplementary Tables S1-S7 These are supplementary tables to provide detailed information about the materials used in the study including cell, culture conditions, growth factors, inhibitors, and antibodies.

Supplementary Figure S1 Combination of SHP099 and α-PD-L1 delays D2.A1 pulmonary growth in vivo

Supplementary Figure S2 The gating ancestry for the populations in the study of D2.A1 model

Supplementary Figure S3 Corresponding representative dot plots for the quantification in figure 2A and additional T cell composition analysis in the study of D2.A1 model.

Supplementary Figure S4 Corresponding representative dot plots for the quantification in figure 2B-C and additional T cell exhaustion marker analysis in the study of D2.A1 model.

Supplementary Figure S5 Corresponding representative dot plots for the quantification in figure 2D-F and additional myeloid composition analysis in the study of D2.A1 model.

Supplementary Figure S6 Depletion of SHP2 in tumor cells delays 4T1 pulmonary metastasis in vivo

Supplementary Figure S7 The gating ancestry for the populations in the study of 4T1 model

Supplementary Figure S8 T cell composition analysis in mice bearing SHP2 manipulated 4T1 metastases and representative dot plots for data shown in figure 4A.

Supplementary Figure S9 Representative dot plots for data shown in figure 4B-C and additional exhaustion marker analysis of CD4+ T cells in mice bearing SHP2 manipulated 4T1 metastases.

Supplementary Figure S10 Representative dot plots for data shown in figure 4D-F and additional exhaustion marker analysis of CD8+ T cells in mice bearing SHP2 manipulated 4T1 metastases.

Supplementary Figure S11 Representative dot plots for data shown in figure 4G-H and additional myeloid composition analysis in mice bearing 4T1 metastases.

Supplementary Figure S12 Analyses of Tregs and macrophage scores in clinical datasets with respect to SHP2 phosphorylation.

Supplementary Figure S13 Inhibition or depletion of SHP2 and PD-L1 blockade rescue T cell cytotoxicity.

Supplementary Figure S14 PD-L1 expression is induced by growth factor stimulation in the D2.A1 cells.

Supplementary Figure S15 Growth factors and 3D culture environment induce PD-L1 in MBC cells.

Supplementary Figure S16 SHP2 regulates MHC class I expression via the balance between MAPK and STAT1 signaling in human MBC cells.
