# Supplementary material for: Tumor Cell–Autonomous SHP2 Contributes to Immune Suppression in Metastatic Breast Cancer
Source: Cancer Res Commun. 2022 Oct 3;2(10):1104–18. doi: 10.1158/2767-9764.CRC-22-0117 (PMC10035406; doi:10.1158/2767-9764.CRC-22-0117)
Supplement: Supplementary Information SI1 — This is the code information for publicly available data analysis. [file crc-22-0117-s01.docx]

**Tumor-cell autonomous SHP2 contributes to immune suppression in metastatic breast cancer**

Hao Chen^1^, Gregory M. Cresswell^2^, Sarah Libring^3^, Mitchell G. Ayers^1^, Jinmin Miao^1^, Zhong-Yin Zhang^1,4^, Luis Solorio^3,4^, Timothy L. Ratliff^2,4^, Michael K. Wendt^1,4,*^

**Supplementary Information**

**Original codes are available on GitHub: https://github.com/benchlover/SHP2_immunology**

Figure 5A and 5B demonstrating the differential immune scores and stroma scores in patients grouped by phosphorylation levels of SHP2 at Y542 or expression levels of SHP2 were generated with Python Code 1.

Figure 5C demonstrating differential phosphorylation levels of SHP2 at Y542 and expression levels of SHP2 in patients grouped by CD4^+^ T-cell infiltration levels was generated with R code 1 and Python Code 2.

Figure 5D demonstrating differential phosphorylation levels of SHP2 at Y542 and expression levels of SHP2 in patients grouped by M1 Macrophage infiltration levels was generated with R code 1 and Python Code 5.

Figure 5E and 5F demonstrating differential gene expression in patients grouped by phosphorylation levels of SHP2 at Y542 or expression levels of SHP2 were generated with Python Code 3.

Figure 5G demonstrating ssGSEA analysis was generated with Python code 4 and R code 2.

Figure 5H and 5I demonstrating GSEA analyses were generated with GSEA 4.1.0. The pathway files were downloaded from GSEA websites (http://www.gsea-msigdb.org/gsea/index.jsp).

Supplementary Figure 11A and B demonstrating differential phosphorylation levels of SHP2 at Y542 and expression levels of SHP2 in patients grouped by Tregs and M2 Macrophage infiltration levels was generated with R code 1 and Python Code 5.

Supplementary Figure 11C demonstrating differential CD8 T-cell-specific gene expression in patients grouped by phosphorylation levels of SHP2 at Y542 was generated with CIBERSORTx (https://cibersortx.stanford.edu/) and corresponding Python code 6.
