# Supplementary material for: Tumor Cell–Autonomous SHP2 Contributes to Immune Suppression in Metastatic Breast Cancer
Source: Cancer Res Commun. 2022 Oct 3;2(10):1104–18. doi: 10.1158/2767-9764.CRC-22-0117 (PMC10035406; doi:10.1158/2767-9764.CRC-22-0117)
Supplement: Supplementary Tables S1-S7 — These are supplementary tables to provide detailed information about the materials used in the study including cell, culture conditions, growth factors, inhibitors, and antibodies. [file crc-22-0117-s02.docx]

**Tumor-cell autonomous SHP2 contributes to immune suppression in metastatic breast cancer**

Hao Chen^1^, Gregory M. Cresswell^2^, Sarah Libring^3^, Mitchell G. Ayers^1^, Jinmin Miao^1^, Zhong-Yin Zhang^1,4^, Luis Solorio^3,4^, Timothy L. Ratliff^2,4^, Michael K. Wendt^1,4,*^

**Supplementary Tables**

**Supplementary Table 1 Cell lines and culture conditions**

| Name of the cell line | Culture condition |
| --- | --- |
| 4T1 (ATCC^®^ CRL-2539™) | DMEM with 10% Fetal Bovine Serum (FBS) |
| D2.A1 | DMEM with 10% Fetal Bovine Serum (FBS) |
| 4TO7 | DMEM with 10% Fetal Bovine Serum (FBS) |
| BT-549 (ATCC^®^ HTB-122™) | RPMI-1640 with 10% Fetal Bovine Serum (FBS) and 0.1% Human Insulin solution |

**Supplementary Table 2 Drugs and reagents for *in vivo* studies**

| Drug / Reagent | Source | Identifier / Clone / formulation |
| --- | --- | --- |
| SHP099 dihydrochloride (*in vivo* grade) | Chemietek | Catalog No: CT-SHP099  Formulation: 0.5% Hydroxypropyl Methylcellulose |
| InVivoMAb anti-mouse PD-L1 (B7-H1) | Bio X Cell | BE0101, Clone 10F.9G2 |
| InVivoMAb rat IgG2b isotype control, anti-keyhole limpet hemocyanin | Bio X Cell | BE0090, Clone LTF-2 |
| Doxycycline Hydrochloride | RPI from Fisher | Catalog No: 50-213-285  Formulation: 2mg/ml in drinking water |
| D-Luciferin, Potassium Salt | GoldBio | Catalog No: LUCK-100 |

**Supplementary Table 3 Antibodies used in flow cytometry for in vivo studies**

| **Antibodies for combination of SHP099 and α-PD-L1 in D2.A1 model** | | | | |
| --- | --- | --- | --- | --- |
| Name | Source | Catalog No | | Panel |
| TruStain FcX™ PLUS (anti-mouse CD16/32) Antibody | Biolegend | 101320 | | Lymphoid & myeloid |
| Zombie Violet™ Fixable Viability Kit | Biolegend | 423114 | | Lymphoid & myeloid |
| PerCP anti-mouse CD45 Antibody | Biolegend | 103129 | | Lymphoid & myeloid |
| FITC anti-mouse CD8a Antibody | Biolegend | 100705 | | lymphoid |
| Brilliant Violet 785™ anti-mouse CD4 Antibody | Biolegend | 100453 | | lymphoid |
| APC anti-mouse CD366 (Tim-3) Antibody | Biolegend | 134007 | | lymphoid |
| PE/Dazzle™ 594 anti-mouse CD279 (PD-1) Antibody | Biolegend | 135227 | | lymphoid |
| PE/Cyanine7 anti-mouse CD223 (LAG-3) Antibody | Biolegend | 125225 | | lymphoid |
| PE/Cyanine7 anti-mouse/human CD11b Antibody | Biolegend | 101215 | | myeloid |
| Brilliant Violet 605™ anti-mouse F4/80 Antibody | Biolegend | 123133 | | myeloid |
| PE anti-mouse CD274 (B7-H1, PD-L1) Antibody | Biolegend | 124307 | | myeloid |
| Brilliant Violet 711™ anti-mouse CD206 (MMR) Antibody | Biolegend | 141727 | | myeloid |
| PE/Dazzle™ 594 anti-mouse CD86 Antibody | Biolegend | 105041 | | myeloid |
| APC/Cyanine7 anti-mouse Ly-6G Antibody | Biolegend | 127623 | | myeloid |
| FITC anti-mouse Ly-6C Antibody | Biolegend | 128005 | | myeloid |
|  | | | |  |
| **Antibodies for doxycycline inducible depletion of SHP2 in 4T1 model** | | | | |
| Name | Source | | Catalog No | Panel |
| TruStain FcX™ PLUS (anti-mouse CD16/32) Antibody | Biolegend | | 101320 | Lymphoid & myeloid |
| Zombie Violet™ Fixable Viability Kit | Biolegend | | 423114 | Lymphoid & myeloid |
| PerCP anti-mouse CD45 Antibody | Biolegend | | 103129 | Lymphoid & myeloid |
| Pacific Blue™ anti-mouse CD8a Antibody | Biolegend | | 100728 | lymphoid |
| Brilliant Violet 711™ anti-mouse CD4 Antibody | Biolegend | | 100447 | lymphoid |
| APC anti-mouse CD366 (Tim-3) Antibody | Biolegend | | 134007 | lymphoid |
| PE/Dazzle™ 594 anti-mouse CD279 (PD-1) Antibody | Biolegend | | 135227 | lymphoid |
| PE/Cyanine7 anti-mouse CD223 (LAG-3) Antibody | Biolegend | | 125225 | lymphoid |
| PE/Cyanine7 anti-mouse/human CD11b Antibody | Biolegend | | 101215 | myeloid |
| Brilliant Violet 605™ anti-mouse F4/80 Antibody | Biolegend | | 123133 | myeloid |
| PE anti-mouse CD274 (B7-H1, PD-L1) Antibody | Biolegend | | 124307 | myeloid |
| Brilliant Violet 711™ anti-mouse CD206 (MMR) Antibody | Biolegend | | 141727 | myeloid |
| PE/Dazzle™ 594 anti-mouse CD86 Antibody | Biolegend | | 105041 | myeloid |
| APC/Cyanine7 anti-mouse Ly-6G Antibody | Biolegend | | 127623 | myeloid |
| APC anti-mouse Ly-6C Antibody | Biolegend | | 128015 | myeloid |

**Supplementary Table 4 Growth factors and inhibitors in T cell killing assays**

| Growth factors / Inhibitors | Source | Identifier |
| --- | --- | --- |
| SHP099 dihydrochloride | Selleck | Catalog No: S8278 |
| TNO155 | Chemietek | Catalog No: CT-TNO155 |
| InVivoMAb anti-mouse PD-L1 (B7-H1) | Bio X Cell | Catalog No: BE0101, Clone 10F.9G2 |
| InVivoMAb rat IgG2b isotype control, anti-keyhole limpet hemocyanin | Bio X Cell | Catalog No: BE0090, Clone LTF-2 |
| Basic FGF (FGF2), Human | GoldBio | Catalog No: 1140-02-10 |
| Recombinant Human PDGF-BB Protein, CF | R&D systems | Catalog No: 220-BB-010 |

**Supplementary Table 5 Growth factors and inhibitors in flow cytometry for MBC cells**

| Growth factors / Inhibitors | Source | Identifier |
| --- | --- | --- |
| SHP099 dihydrochloride | Selleck | Catalog No: S8278 |
| TNO155 | Chemietek | Catalog No: CT-TNO155 |
| PP2 | Selleck | Catalog No: S7008 |
| PF-562,271 (PF271) | Pfizer | Under agreement from Pfizer Inc. |
| Trametinib | Selleck | Catalog No: S2673 |
| Alpelisib | Selleck | Catalog No: S2814 |
| Basic FGF (FGF2), Human | GoldBio | Catalog No: 1140-02-10 |
| Recombinant Human PDGF-BB Protein, CF | R&D systems | Catalog No: 220-BB-010 |
| Recombinant Mouse HGF Protein, CF | R&D systems | Catalog No: 2207-HG/CF |
| Recombinant Mouse VEGF 164 Protein, CF | R&D systems | Catalog No: 493-MV-005/CF |
| EGF, Human | GoldBio | Catalog No: 1150-04-100 |
| Human interferon-γ | Peprotech | Catalog No: 300-02 |
| Mouse interferon-γ | Peprotech | Catalog No: 315-05 |

**Supplementary Table 6 Antibodies used in flow cytometry for MBC cells**

| Antibody | Source | Catalog No. |
| --- | --- | --- |
| PE anti-mouse CD274 (B7-H1, PD-L1) Antibody | Biolegend | 124307 |
| FITC anti-mouse H-2 Antibody | Biolegend | 125508 |
| PerCP/Cyanine5.5 anti-human CD274 (B7-H1, PD-L1) Antibody | Biolegend | 329737 |
| PE anti-human HLA-A,B,C Antibody | Biolegend | 311405 |

**Supplementary Table 7 Primary and secondary antibodies**

| Antibody | Source | Identifier | Host |
| --- | --- | --- | --- |
| Phospho-Stat1 (Tyr701) (58D6) Rabbit mAb | Cell Signaling Technology | Catalog No: #9167 | Rabbit |
| Stat1 Antibody | Cell Signaling Technology | Catalog No: #9172 | Rabbit |
| Phospho-p44/42 MAPK (Erk1/2) (Thr202/Tyr204) Antibody | Cell Signaling Technology | Catalog No: #9101 | Rabbit |
| p44/42 MAPK (Erk1/2) Antibody | Cell Signaling Technology | Catalog No: #9102 | Rabbit |
| Tubulin, beta | Developmental Studies Hybridoma Bank (DSHB) | Catalog No: E7 | Mouse |
| Goat anti-Mouse IgG (H+L) Secondary Antibody, HRP | ThermoFisher | Catalog No: 62-6520 | Goat |
| Goat anti-Rabbit IgG (H+L) Secondary Antibody, HRP | ThermoFisher | Catalog No: 65-6120 | Goat |
| IRDye^®^ 680RD Goat anti-Mouse IgG Secondary Antibody | LI-COR, Inc. | P/N No: 926-68070 | Goat |
| IRDye® 800CW Goat anti-Rabbit IgG Secondary Antibody | LI-COR, Inc. | P/N No: 926-32211 | Goat |
