# Supplementary material for: Tumor Cell–Autonomous SHP2 Contributes to Immune Suppression in Metastatic Breast Cancer
Source: Cancer Res Commun. 2022 Oct 3;2(10):1104–18. doi: 10.1158/2767-9764.CRC-22-0117 (PMC10035406; doi:10.1158/2767-9764.CRC-22-0117)
Supplement: Supplementary Figure S1 — Combination of SHP099 and α-PD-L1 delays D2.A1 pulmonary growth in vivo [file crc-22-0117-s03.pdf]

Supplementary Figure 1

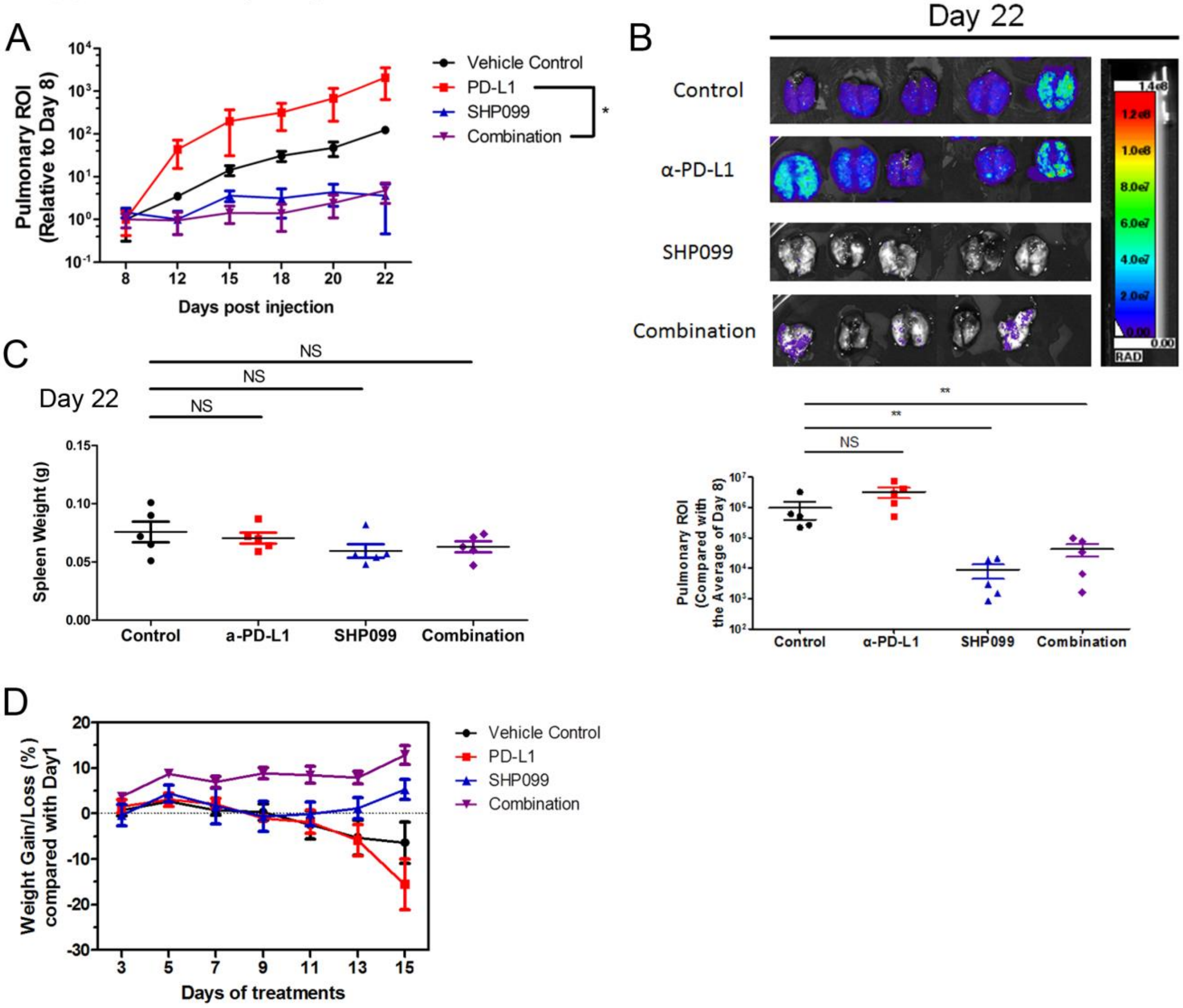

Supplementary Figure 1. Combination of SHP099 and α-PD-L1 delays D2.A1 pulmonary growth *in vivo* A, Bioluminescence values for pulmonary regions of interest (ROI) from mice with D2.A1 cell growth in lungs normalized to Day 8 post injection. Data are the mean ± s.e.m. of 5 mice per treatment group. *p<0.05. B, (top) Representative bioluminescent images of the lungs at Day 22 post injection and (bottom) quantified ROI values of the lungs normalized to the average ROI values of Day 8 post injection. Data are the individual plots and mean ± s.e.m. of 5 mice per group, NS: not significant, **p<0.01 determined by Mann Whitney test. C, Dot plot showing the spleen weight of the mice at Day 22 post injection. Data are the individual plots and mean ± s.e.m. of 5 mice per group, NS: not significant determined by Mann Whitney test. D, Mice were treated with SHP099 and α-PD-L1 as indicated in figure 1. The body weights of mice were monitored every 2 days, and the percentage of weight gain/loss was normalized to Day 1 of the treatment.
