# Supplementary material for: Tumor Cell–Autonomous SHP2 Contributes to Immune Suppression in Metastatic Breast Cancer
Source: Cancer Res Commun. 2022 Oct 3;2(10):1104–18. doi: 10.1158/2767-9764.CRC-22-0117 (PMC10035406; doi:10.1158/2767-9764.CRC-22-0117)
Supplement: Supplementary Figure S2 — The gating ancestry for the populations in the study of D2.A1 model [file crc-22-0117-s04.pdf]

Supplementary Figure 2

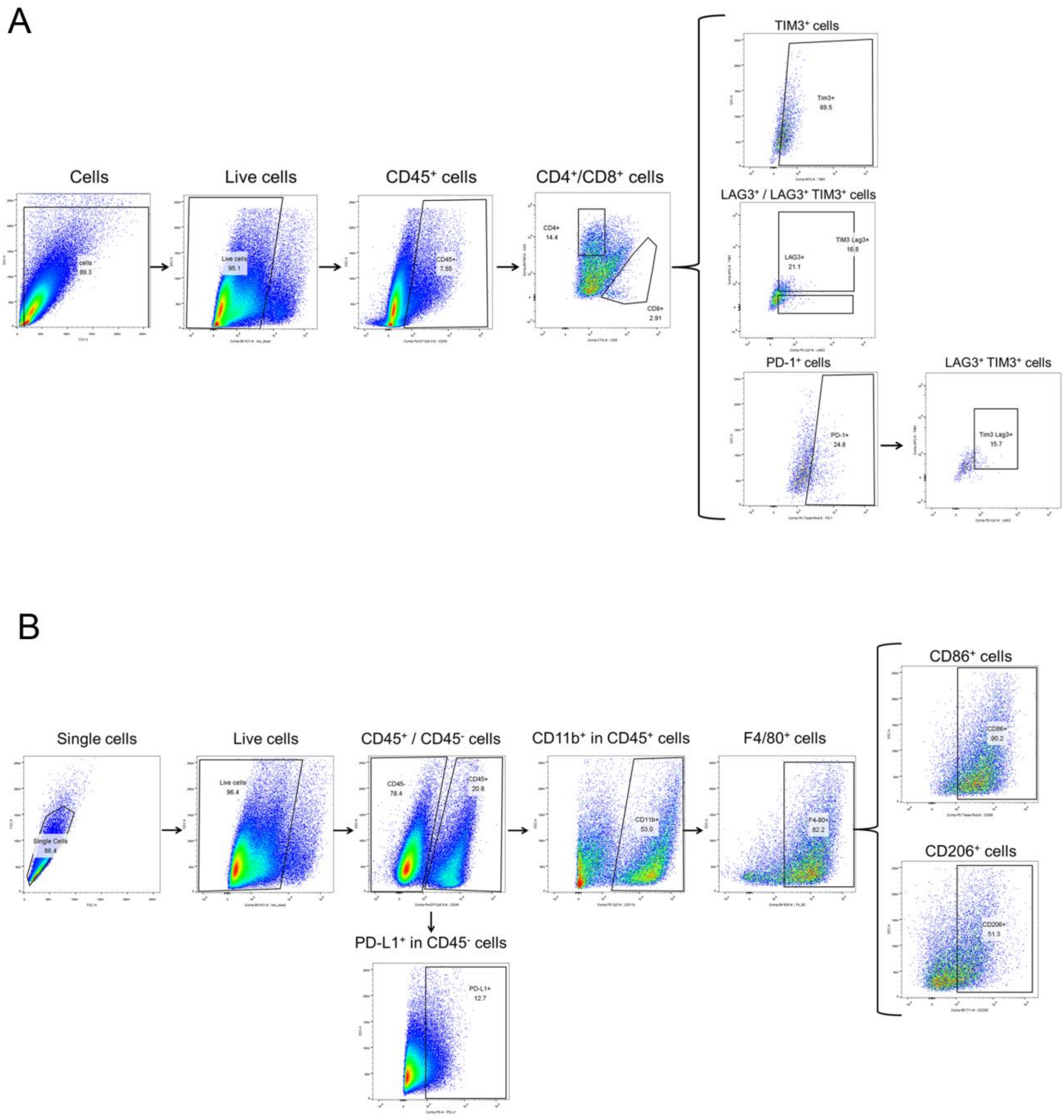

Supplementary Figure 2. The gating ancestry for the populations in the study of D2.A1 model A, Spleens and tumors from lung tissues were isolated and digested into single cell suspensions, and stained with lymphoid antibody panel as described in the materials and methods. These cells were sequentially gated as shown to identify a population of cells, live cells, $CD45^+$ and $CD4^+$/$CD8^+$ cells prior to analysis of $TIM3^+$, $LAG3^+$, $TIM3^+LAG3^+$ and PD-1^+. The $TIM3^+LAG3^+$ cells were also gated under PD-1^+ population. B, Tumors from lung tissues were isolated and digested into single cell suspensions, and stained with myeloid antibody panel as described in the materials and methods. These cells were sequentially gated as shown to identify a population of single cells, live cells, $CD45^+$/$CD45^-$, $CD11b^+$ and F4/80^+ cells prior to analysis of $CD86^+$ and $CD206^+$. The PD-$L1^+$ cells were also gated under $CD45^-$ population.
