# Supplementary material for: Tumor Cell–Autonomous SHP2 Contributes to Immune Suppression in Metastatic Breast Cancer
Source: Cancer Res Commun. 2022 Oct 3;2(10):1104–18. doi: 10.1158/2767-9764.CRC-22-0117 (PMC10035406; doi:10.1158/2767-9764.CRC-22-0117)
Supplement: Supplementary Figure S3 — Corresponding representative dot plots for the quantification in figure 2A and additional T cell composition analysis in the study of D2.A1 model. [file crc-22-0117-s05.pdf]

Supplementary Figure 3

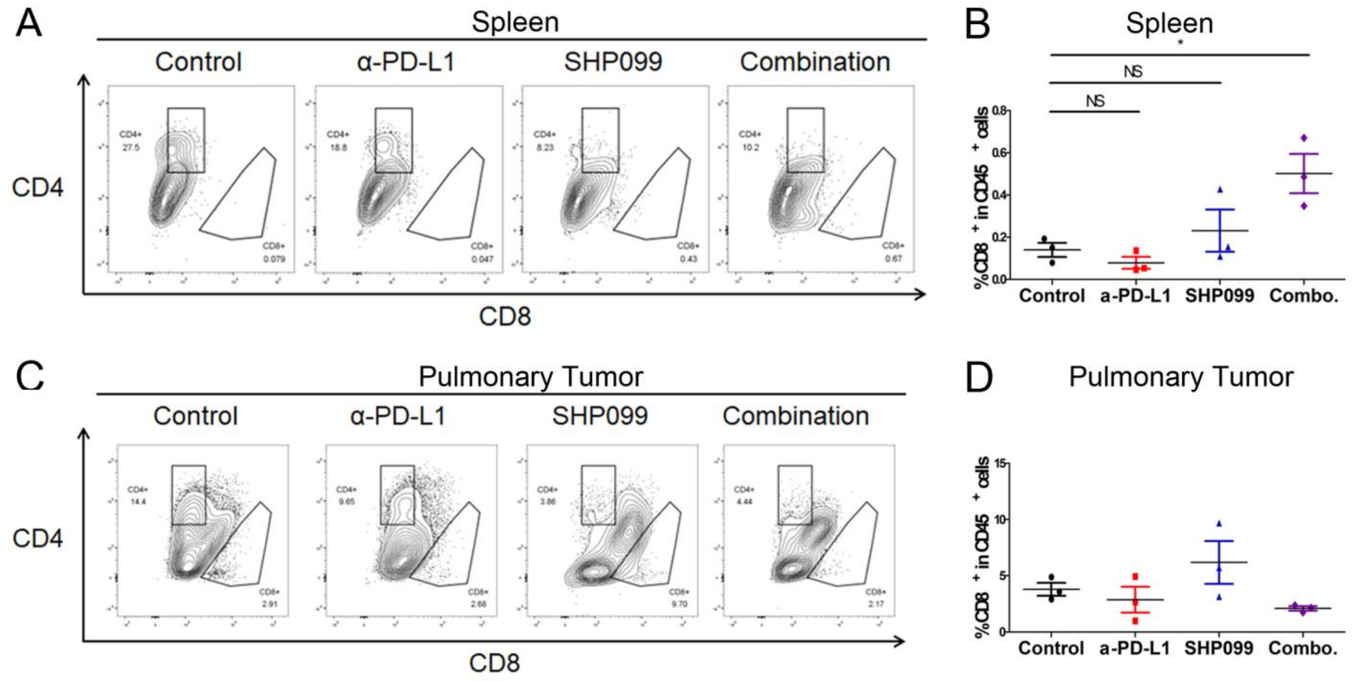

Supplementary Figure 3. Corresponding representative dot plots for the quantification in figure 2A and additional T cell composition analysis in the study of D2.A1 model. A, Representative dot plots of $CD4^+$ and $CD8^+$ population as a frequency of $CD45^+$ cells in isolated spleens of each group. B, Quantification of $CD8^+$ populations as a frequency of $CD45^+$ cells in isolated spleens of each group. NS: not significant, *p<0.05, n=3. C, Representative dot plots of $CD4^+$ and $CD8^+$ population as a frequency of $CD45^+$ cells in isolated lung tissues (right) of each group. D, Quantification of $CD8^+$ populations as a frequency of $CD45^+$ cells in isolated lung tissues (right) of each group.
