# Supplementary material for: Tumor Cell–Autonomous SHP2 Contributes to Immune Suppression in Metastatic Breast Cancer
Source: Cancer Res Commun. 2022 Oct 3;2(10):1104–18. doi: 10.1158/2767-9764.CRC-22-0117 (PMC10035406; doi:10.1158/2767-9764.CRC-22-0117)
Supplement: Supplementary Figure S4 — Corresponding representative dot plots for the quantification in figure 2B-C and additional T cell exhaustion marker analysis in the study of D2.A1 model. [file crc-22-0117-s06.pdf]

Supplementary Figure 4

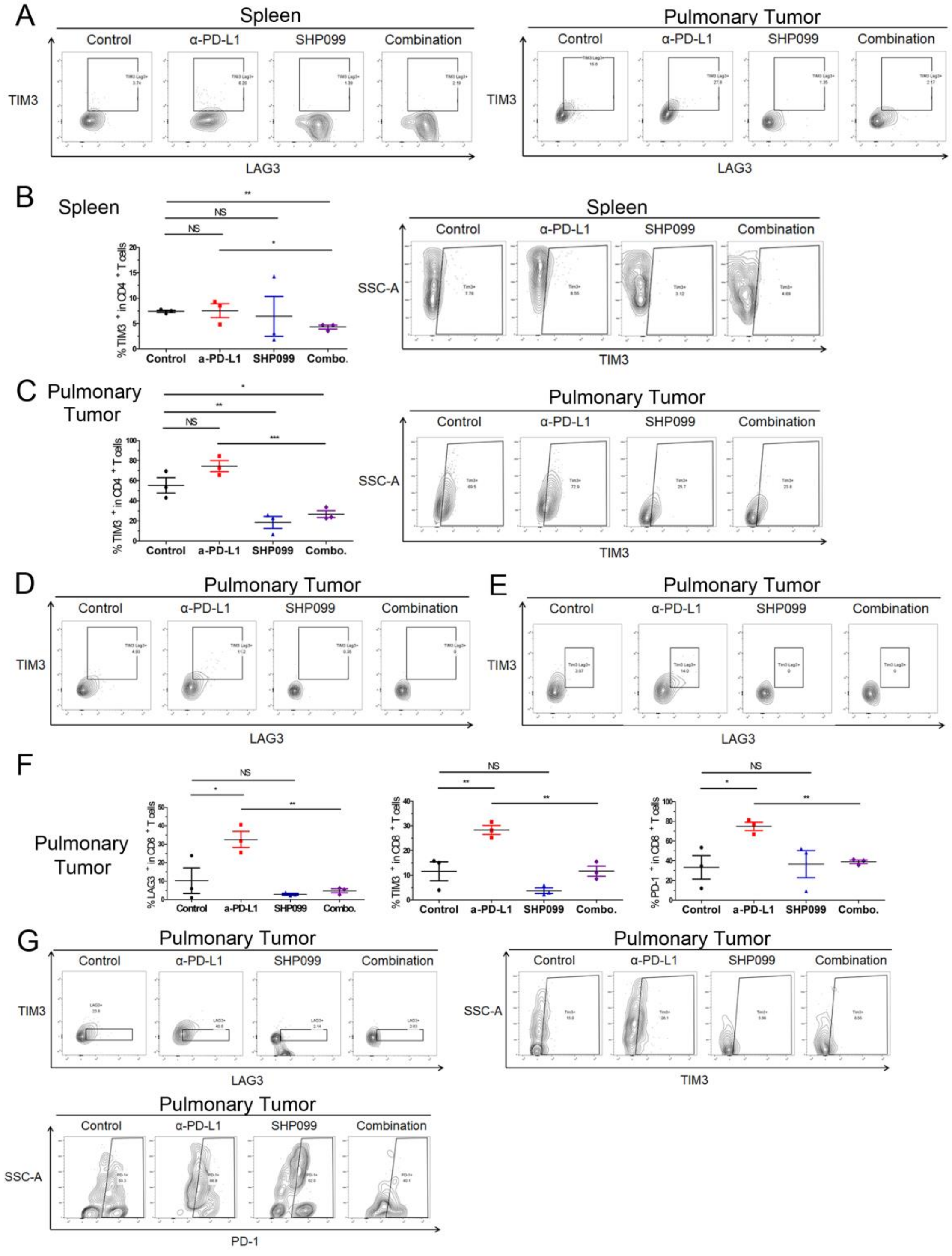

Supplementary Figure 4. Corresponding representative dot plots for the quantification in figure 2B-C and additional T cell exhaustion marker analysis in the study of D2.A1 model. A, Representative dot plots of $TIM3^+LAG3^+$ population as a frequency of $CD45^+CD4^+$ cells in isolated spleens (left) and lung tissues (right) of each group. B, C, Quantification and representative dot plots of $TIM3^+$ population as a frequency of $CD45^+CD4^+$ cells in isolated spleens (B) and lung tissues (C) of each group. D,E, Representative dot plots of $TIM3^+LAG3^+$ population as a frequency of $CD45^+CD8^+$ cells (D) and $CD45^+CD8^+PD\text{-}1^+$ cells (E) of in isolated lung tissues of each group. F, Quantification of $LAG3^+$ (left), $TIM3^+$ (middle) and $PD\text{-}1^+$ (right) population as a frequency of $CD45^+CD8^+$ cells in isolated lung tissues of each group. G, Representative dot plots of the quantification in F. In all panels, NS: not significant, *$p<0.05$, **$p<0.01$, ***$p<0.001$, n=3.
