# Supplementary material for: Tumor Cell–Autonomous SHP2 Contributes to Immune Suppression in Metastatic Breast Cancer
Source: Cancer Res Commun. 2022 Oct 3;2(10):1104–18. doi: 10.1158/2767-9764.CRC-22-0117 (PMC10035406; doi:10.1158/2767-9764.CRC-22-0117)
Supplement: Supplementary Figure S5 — Corresponding representative dot plots for the quantification in figure 2D-F and additional myeloid composition analysis in the study of D2.A1 model. [file crc-22-0117-s07.pdf]

Supplementary Figure 5

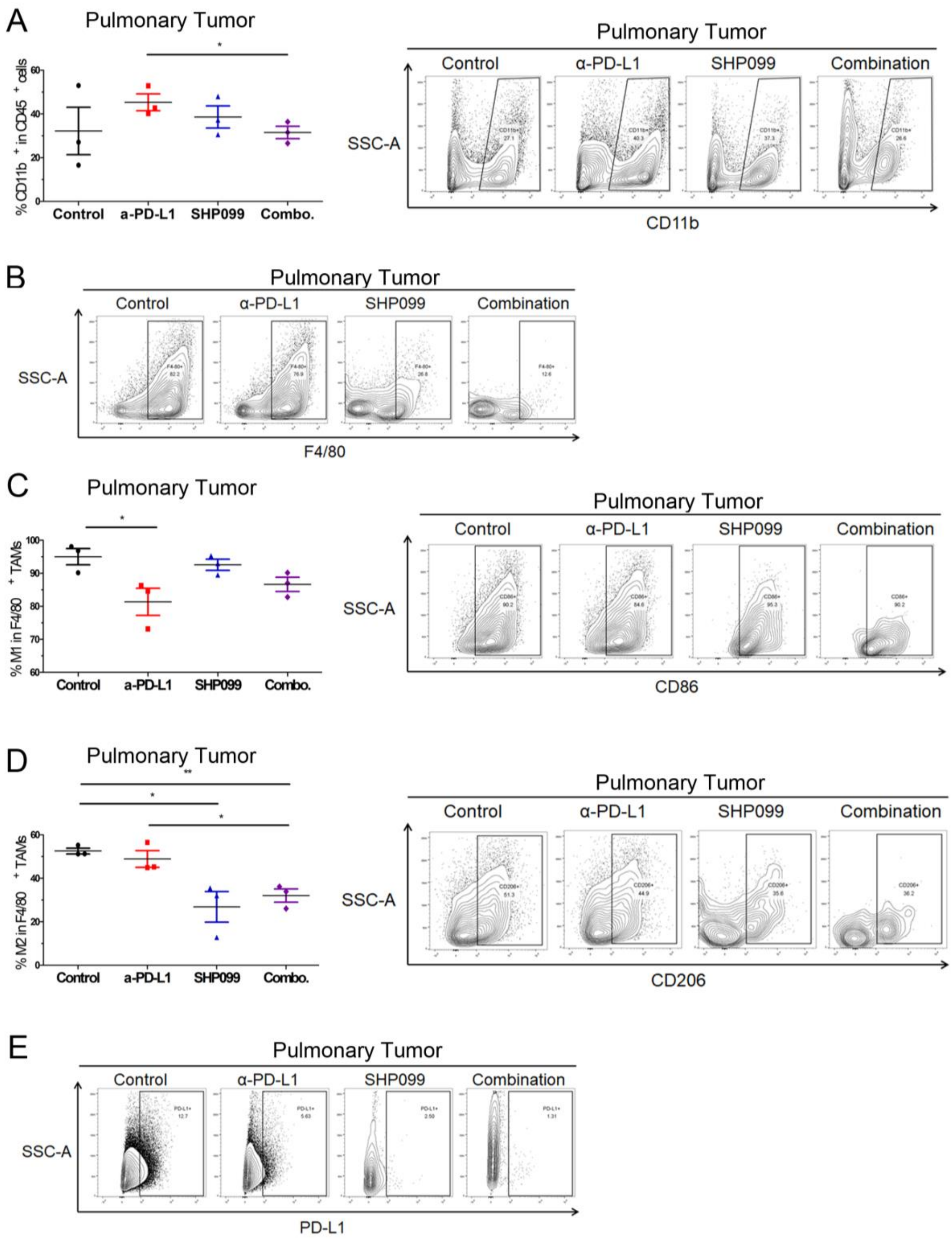

Supplementary Figure 5. Corresponding representative dot plots for the quantification in figure 2D-F and additional myeloid composition analysis in the study of D2.A1 model. A, Representative dot plots and quantification of $CD11b^+$ population as a frequency of $CD45^+$ cells in isolated lung tissues of each group. B, Representative dot plots of $F4/80^+$ population as a frequency of $CD45^+CD11b^+$ cells in isolated lung tissues of each group. C, D, Representative dot plots and quantification of $CD86^+$ (C) and $CD206^+$ (D) population as a frequency of $CD45^+CD11b^+F4/80^+$ cells in isolated lung tissues of each group. In all panels, *p<0.05, **p<0.01, n=3. E, Representative dot plots of $PD\text{-}L1^+$ population as a frequency of $CD45^-$ cells in isolated lung tissues of each group.
