# Supplementary material for: Tumor Cell–Autonomous SHP2 Contributes to Immune Suppression in Metastatic Breast Cancer
Source: Cancer Res Commun. 2022 Oct 3;2(10):1104–18. doi: 10.1158/2767-9764.CRC-22-0117 (PMC10035406; doi:10.1158/2767-9764.CRC-22-0117)
Supplement: Supplementary Figure S6 — Depletion of SHP2 in tumor cells delays 4T1 pulmonary metastasis in vivo [file crc-22-0117-s08.pdf]

Supplementary Figure 6

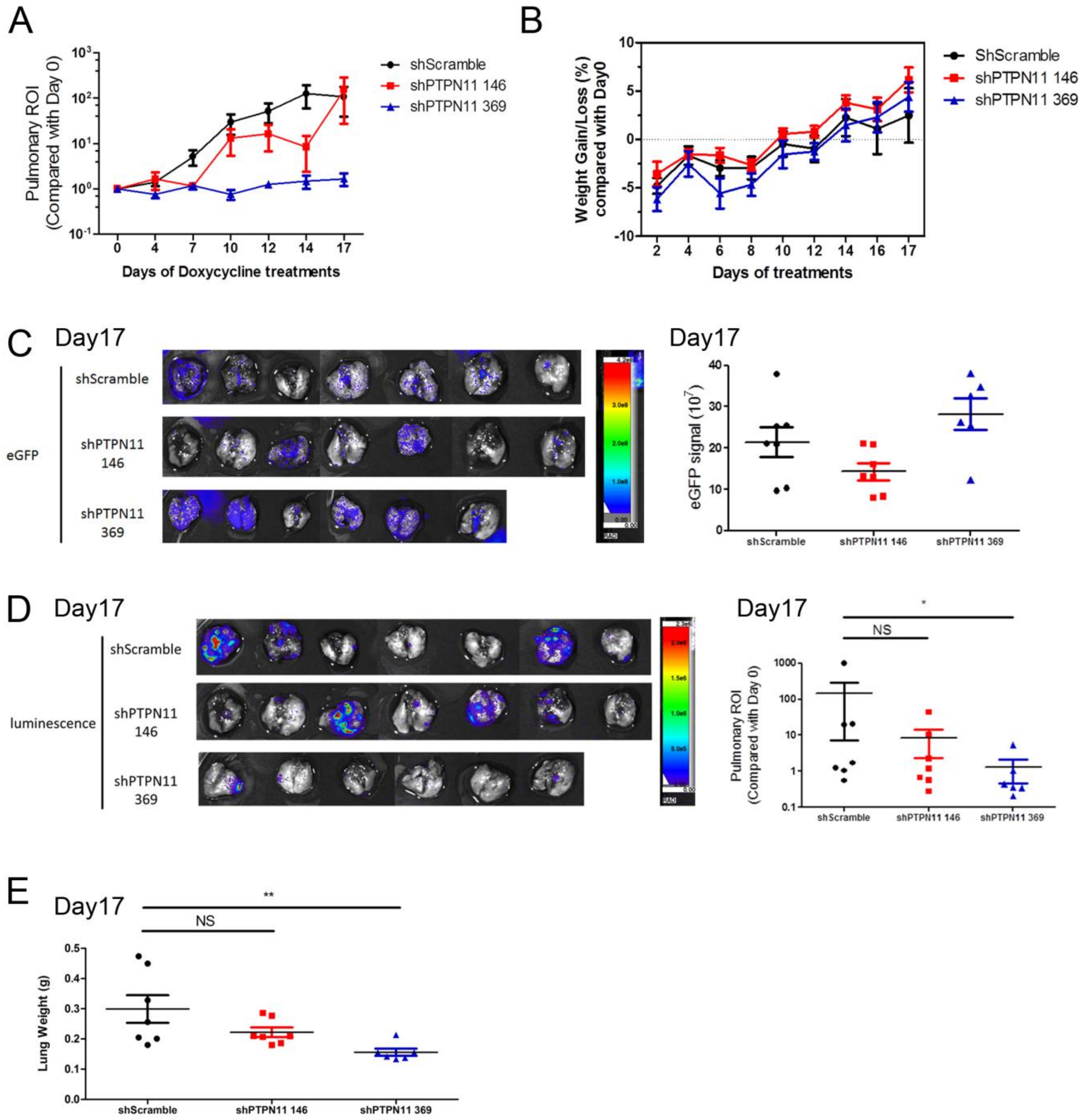

Supplementary Figure 6. Depletion of SHP2 in tumor cells delays 4T1 pulmonary metastasis *in vivo* A, Bioluminescence values for pulmonary regions of interest (ROI) from mice with 4T1 metastatic growth in lungs normalized to Day 0 of doxycycline treatments. Data are the mean ± s.e.m. of n = 7 mice per group for shScramble and shPTPN11 146, n=6 mice per group for shPTPN11 369. B, Mice were treated with doxycycline as indicated in figure 3. The body weights of mice were monitored every 2 days, and the percentage of weight gain/loss was normalized to Day 0 of the treatments. C, (left) Representative Green Fluorescence (eGFP) images of the lungs at Day 17 of doxycycline treatments and (right) quantified eGFP values of the lungs. D, (left) Representative bioluminescent images of the lungs at Day 17 of doxycycline treatments and (right) quantified ROI values of the lungs normalized to the average ROI values of Day 0 of doxycycline treatments. NS: not significant, *p<0.05. E, Plots comparing the wet lung weights of the mice at Day 17 of doxycycline treatments. NS: not significant, **p<0.01.
