# Supplementary material for: Tumor Cell–Autonomous SHP2 Contributes to Immune Suppression in Metastatic Breast Cancer
Source: Cancer Res Commun. 2022 Oct 3;2(10):1104–18. doi: 10.1158/2767-9764.CRC-22-0117 (PMC10035406; doi:10.1158/2767-9764.CRC-22-0117)
Supplement: Supplementary Figure S7 — The gating ancestry for the populations in the study of 4T1 model [file crc-22-0117-s09.pdf]

Supplementary Figure 7

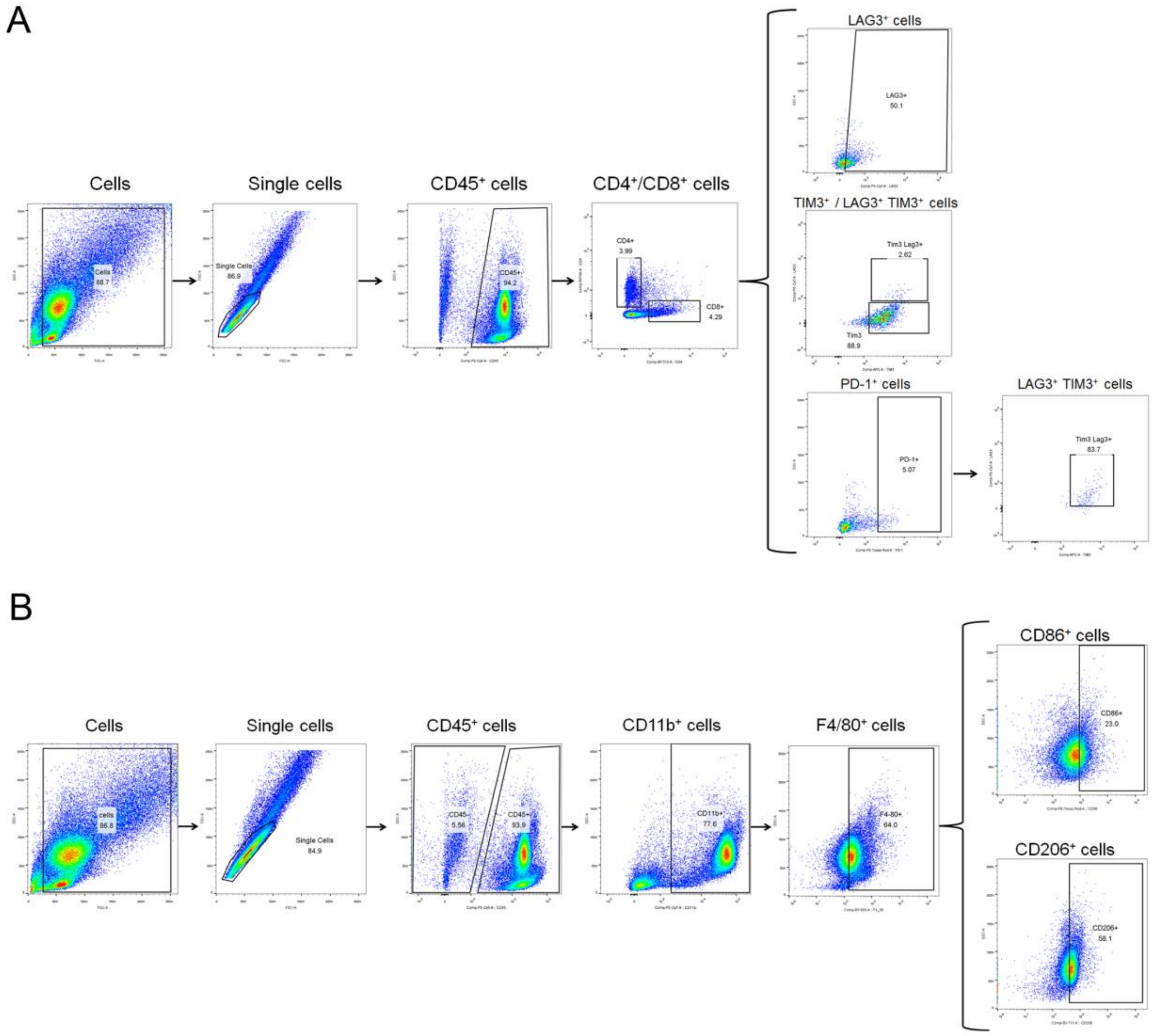

Supplementary Figure 7. The gating ancestry for the populations in the study of 4T1 model A, Spleens and tumors from lung tissues were isolated and digested into single cell suspensions, and stained with lymphoid antibody panel as described in the materials and methods. These cells were sequentially gated as shown to identify a population of cells, single cells, $CD45^+$ and $CD4^+/CD8^+$ cells prior to analysis of $TIM3^+$, $LAG3^+$, $TIM3^+LAG3^+$ and $PD\text{-}1^+$. The $TIM3^+LAG3^+$ cells were also gated under $PD\text{-}1^+$ population. B, Tumors from lung tissues were isolated and digested into single cell suspensions, and stained with myeloid antibody panel as described in the materials and methods. These cells were sequentially gated as shown to identify a population of cells, single cells, $CD45^+$, $CD11b^+$ and $F4/80^+$ cells prior to analysis of $CD86^+$ and $CD206^+$.
