# Supplementary material for: Tumor Cell–Autonomous SHP2 Contributes to Immune Suppression in Metastatic Breast Cancer
Source: Cancer Res Commun. 2022 Oct 3;2(10):1104–18. doi: 10.1158/2767-9764.CRC-22-0117 (PMC10035406; doi:10.1158/2767-9764.CRC-22-0117)
Supplement: Supplementary Figure S8 — T cell composition analysis in mice bearing SHP2 manipulated 4T1 metastases and representative dot plots for data shown in figure 4A. [file crc-22-0117-s10.pdf]

Supplementary Figure 8

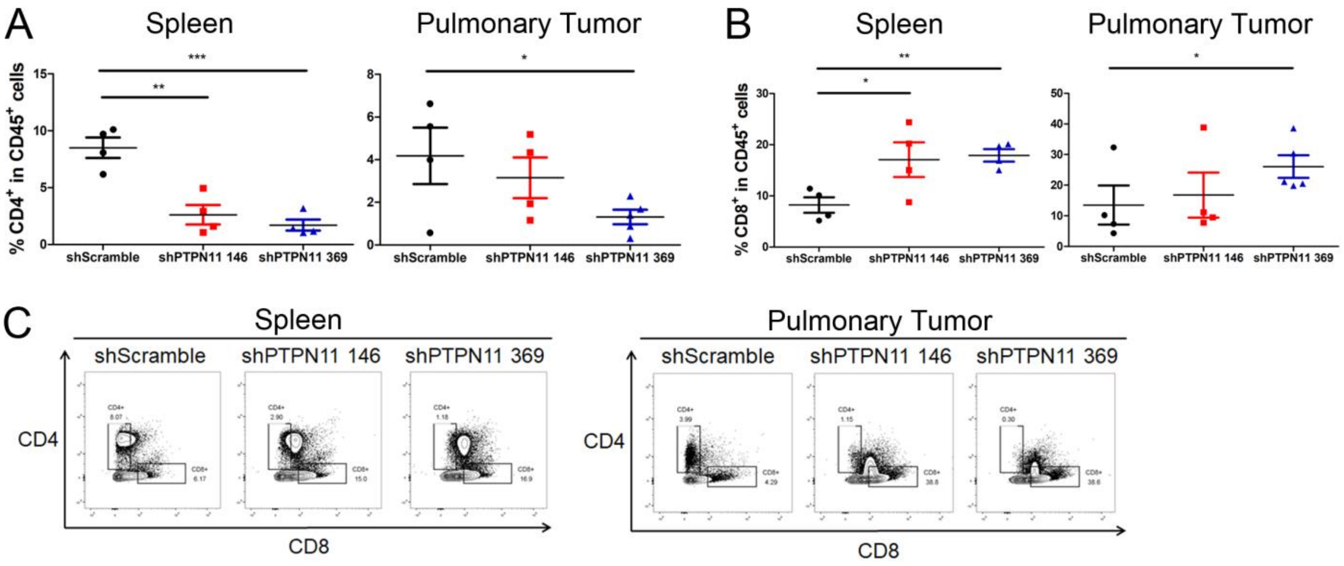

Supplementary Figure 8. T cell composition analysis in mice bearing SHP2 manipulated 4T1 metastases and representative dot plots for data shown in figure 4A. A, B, Quantification of $CD4^+$ (A) and $CD8^+$ (B) population as a frequency of $CD45^+$ cells in isolated spleens (left) and lung tissues (right) of each group. In all panels, *p<0.05, **p<0.01, ***p<0.001; n = 4 for shScramble and shPTPN11 146, n = 5 for shPTPN11 369 in Pulmonary Tumor panels, n=4 for each group in Spleen panels. C, Representative dot plots of $CD4^+$ and $CD8^+$ population as a frequency of $CD45^+$ cells in isolated spleens (left) and lung tissues (right) of each group.
