# Supplementary material for: Tumor Cell–Autonomous SHP2 Contributes to Immune Suppression in Metastatic Breast Cancer
Source: Cancer Res Commun. 2022 Oct 3;2(10):1104–18. doi: 10.1158/2767-9764.CRC-22-0117 (PMC10035406; doi:10.1158/2767-9764.CRC-22-0117)
Supplement: Supplementary Figure S9 — Representative dot plots for data shown in figure 4B-C and additional exhaustion marker analysis of CD4+ T cells in mice bearing SHP2 manipulated 4T1 metastases. [file crc-22-0117-s11.pdf]

Supplementary Figure 9

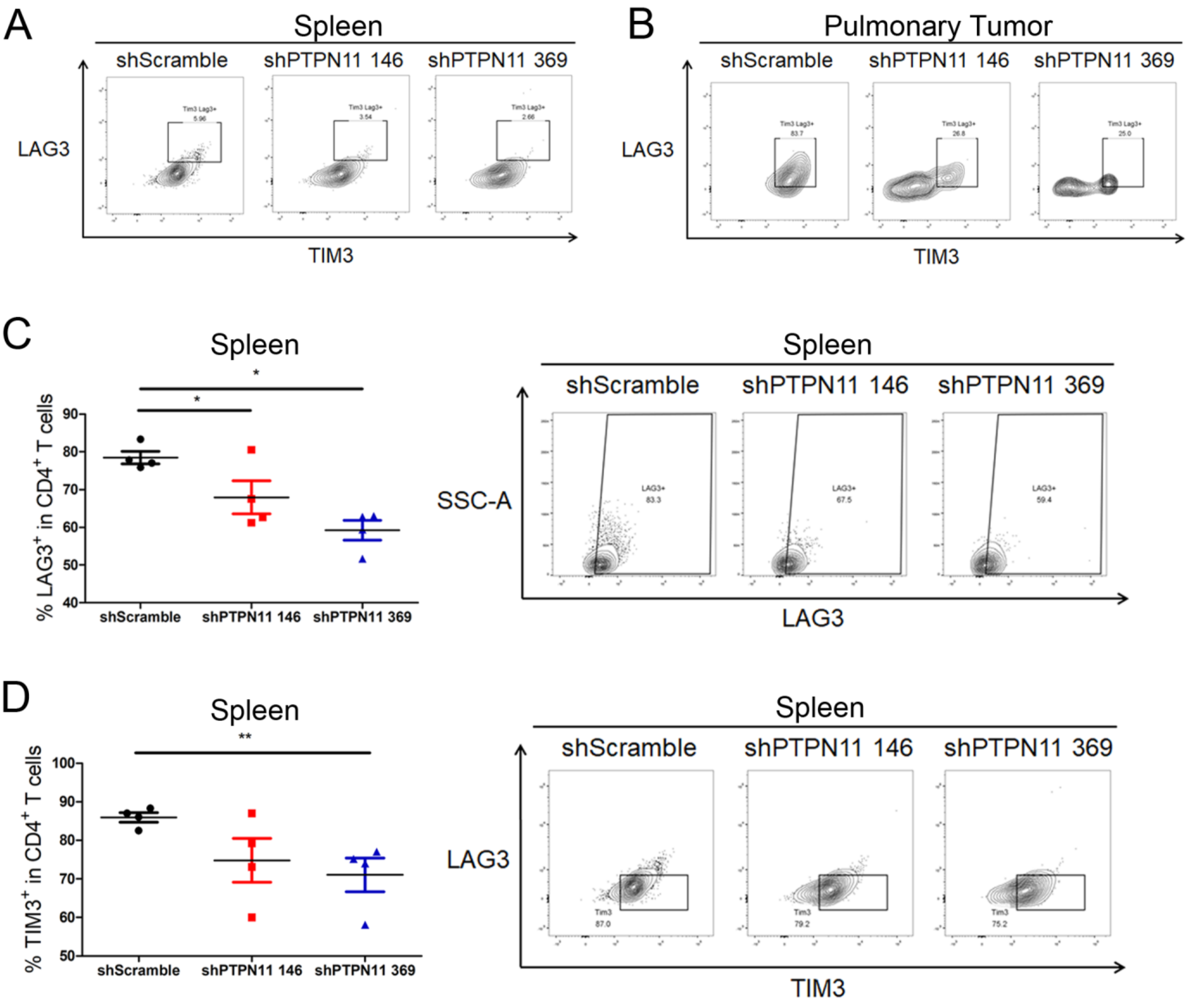

Supplementary Figure 9. Representative dot plots for data shown in figure 4B-C and additional exhaustion marker analysis of CD4$^+$ T cells in mice bearing SHP2 manipulated 4T1 metastases. A, Representative dot plots of TIM3$^+$LAG3$^+$ population as a frequency of CD45$^+$CD4$^+$ cells in isolated spleens of each group. B, Representative dot plots of TIM3$^+$LAG3$^+$ population as a frequency of CD45$^+$CD4$^+$PD-1$^+$ cells in isolated lung tissues of each group. C, D, Representative dot plots and quantification of LAG3$^+$ (C) and TIM3$^+$ (D) population as a frequency of CD45$^+$CD4$^+$ cells in isolated spleens of each group. In all panels, *$p<0.05$, **$p<0.01$, n=4.
