# Supplementary material for: Tumor Cell–Autonomous SHP2 Contributes to Immune Suppression in Metastatic Breast Cancer
Source: Cancer Res Commun. 2022 Oct 3;2(10):1104–18. doi: 10.1158/2767-9764.CRC-22-0117 (PMC10035406; doi:10.1158/2767-9764.CRC-22-0117)
Supplement: Supplementary Figure S10 — Representative dot plots for data shown in figure 4D-F and additional exhaustion marker analysis of CD8+ T cells in mice bearing SHP2 manipulated 4T1 metastases. [file crc-22-0117-s12.pdf]

Supplementary Figure 10

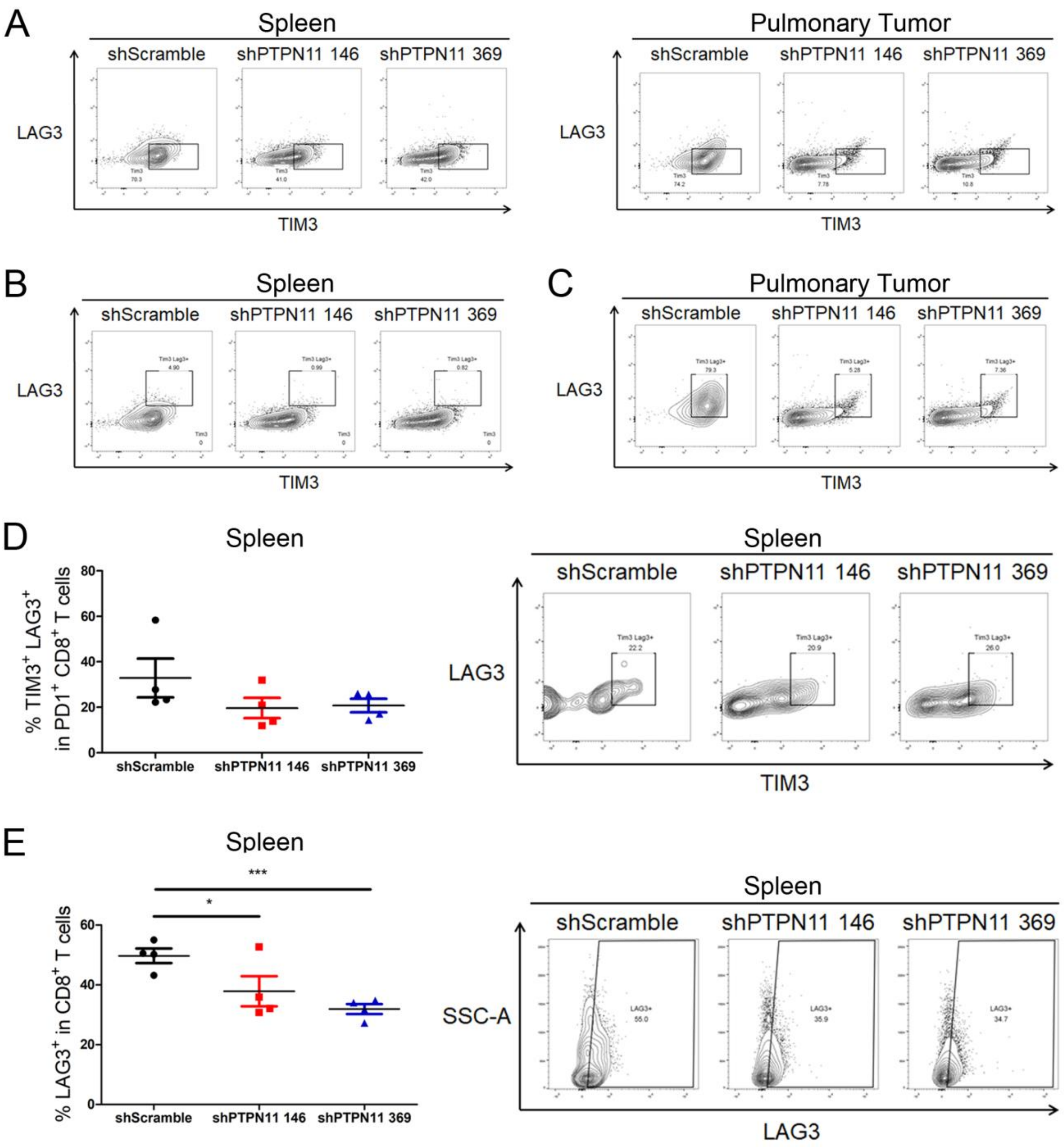

Supplementary Figure 10. Representative dot plots for data shown in figure 4D-F and additional exhaustion marker analysis of CD8$^+$ T cells in mice bearing SHP2 manipulated 4T1 metastases. A, Representative dot plots of TIM3$^+$ population as a frequency of CD45$^+$CD8$^+$ cells in isolated spleens (left) and lung tissues (right) of each group. B, Representative dot plots of TIM3$^+$LAG3$^+$ population as a frequency of CD45$^+$CD8$^+$ cells in isolated spleens of each group. C, Representative dot plots of TIM3$^+$LAG3$^+$ population as a frequency of CD45$^+$CD8$^+$PD-1$^+$ cells in isolated lung tissues of each group. D, Representative dot plots and quantification of TIM3$^+$LAG3$^+$ population as a frequency of CD45$^+$CD8$^+$PD-1$^+$ cells in isolated spleens of each group. E, Representative dot plots and quantification of LAG3$^+$ population as a frequency of CD45$^+$CD8$^+$ cells in isolated spleens of each group. In all panels, *$p<0.05$, ***$p<0.001$, n = 4.
