# Supplementary material for: Tumor Cell–Autonomous SHP2 Contributes to Immune Suppression in Metastatic Breast Cancer
Source: Cancer Res Commun. 2022 Oct 3;2(10):1104–18. doi: 10.1158/2767-9764.CRC-22-0117 (PMC10035406; doi:10.1158/2767-9764.CRC-22-0117)
Supplement: Supplementary Figure S11 — Representative dot plots for data shown in figure 4G-H and additional myeloid composition analysis in mice bearing 4T1 metastases. [file crc-22-0117-s13.pdf]

# Supplementary Figure 11

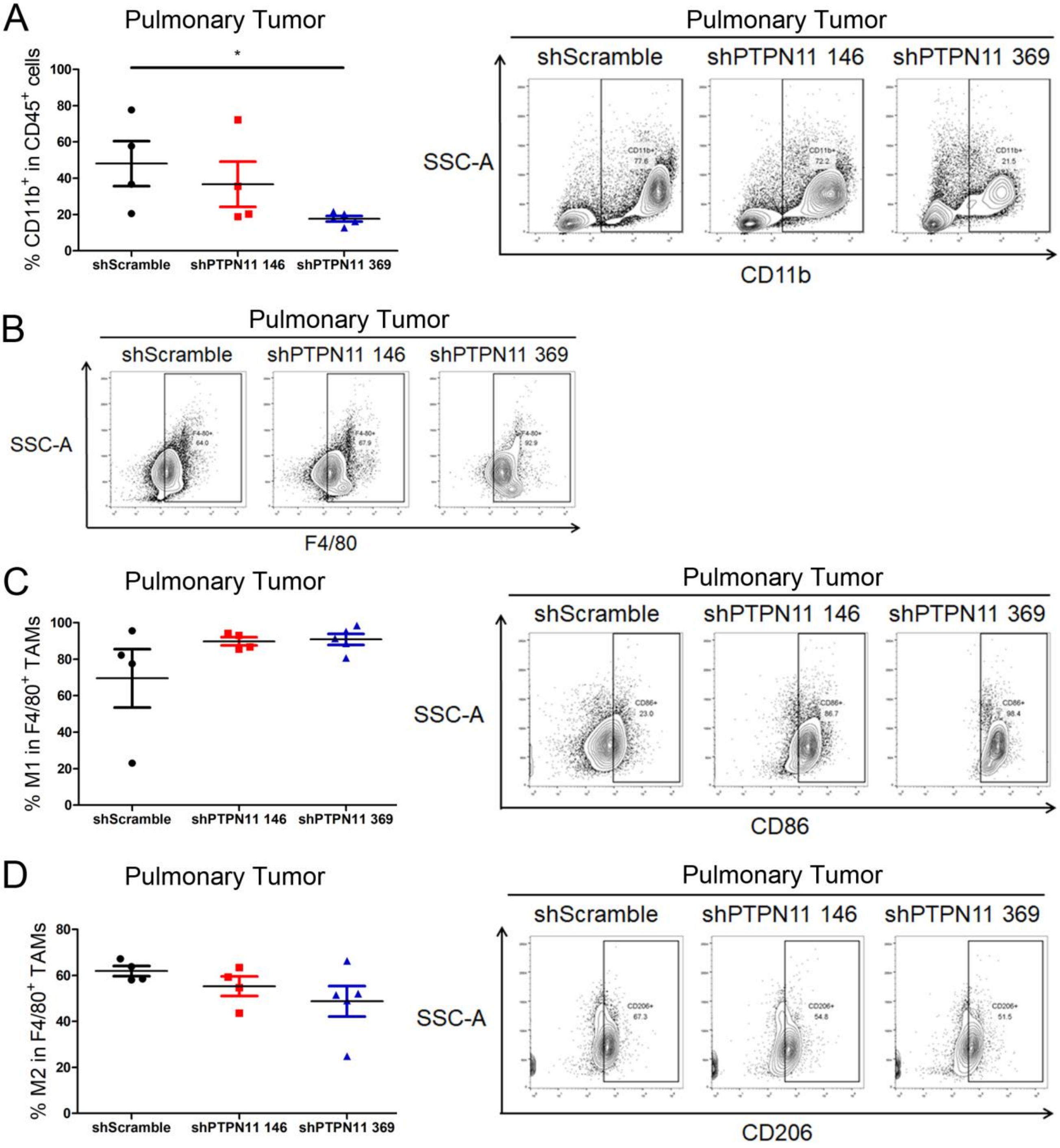


**Supplementary Figure 11. Representative dot plots for data shown in figure 4G-H and additional myeloid composition analysis in mice bearing 4T1 metastases.** A, Representative dot plots and quantification of $CD11b^+$ population as a frequency of $CD45^+$ cells in isolated lung tissues of each group. B, Representative dot plots of $F4/80^+$ population as a frequency of $CD45^+CD11b^+$ cells in isolated lung tissues of each group. C, D, Representative dot plots and quantification of $CD86^+$ (C) and $CD206^+$ (D) population as a frequency of $CD45^+CD11b^+F4/80^+$ cells in isolated lung tissues of each group. In all panels, *$p<0.05$, n = 4 for shScramble and shPTPN11 146, n = 5 for shPTPN11 369.
