# Supplementary material for: Tumor Cell–Autonomous SHP2 Contributes to Immune Suppression in Metastatic Breast Cancer
Source: Cancer Res Commun. 2022 Oct 3;2(10):1104–18. doi: 10.1158/2767-9764.CRC-22-0117 (PMC10035406; doi:10.1158/2767-9764.CRC-22-0117)
Supplement: Supplementary Figure S12 — Analyses of Tregs and macrophage scores in clinical datasets with respect to SHP2 phosphorylation. [file crc-22-0117-s14.pdf]

Supplementary Figure 12

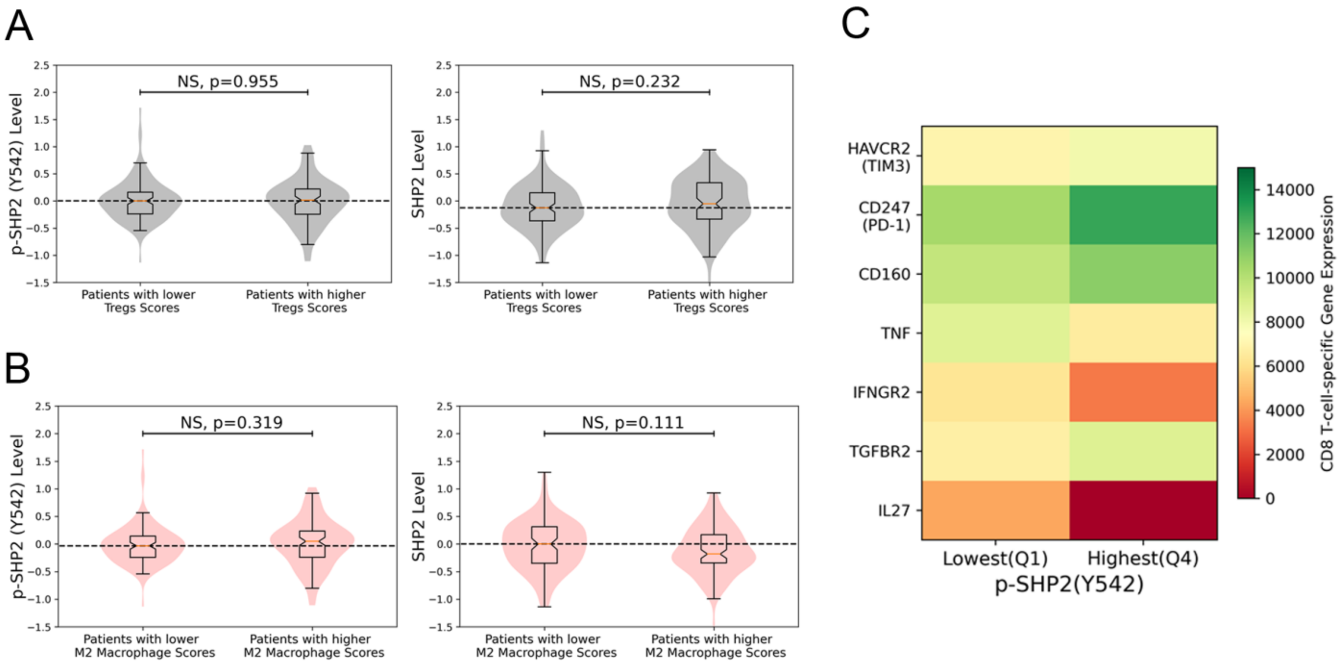

Supplementary Figure 12. Analyses of Tregs and macrophage scores in clinical datasets with respect to SHP2 phosphorylation. A, B, Violin and box plots comparing the differential phosphorylation levels of SHP2 at Y542 and expression levels of SHP2 in patients grouped by Tregs infiltration (A) and M2 Macrophage infiltration (B) levels. C, Heat maps comparing the differential CD8 T-cell-specific gene expression in patients grouped by phosphorylation levels of SHP2 at Y542.
