# Supplementary material for: Tumor Cell–Autonomous SHP2 Contributes to Immune Suppression in Metastatic Breast Cancer
Source: Cancer Res Commun. 2022 Oct 3;2(10):1104–18. doi: 10.1158/2767-9764.CRC-22-0117 (PMC10035406; doi:10.1158/2767-9764.CRC-22-0117)
Supplement: Supplementary Figure S13 — Inhibition or depletion of SHP2 and PD-L1 blockade rescue T cell cytotoxicity. [file crc-22-0117-s15.pdf]

Supplementary Figure 13

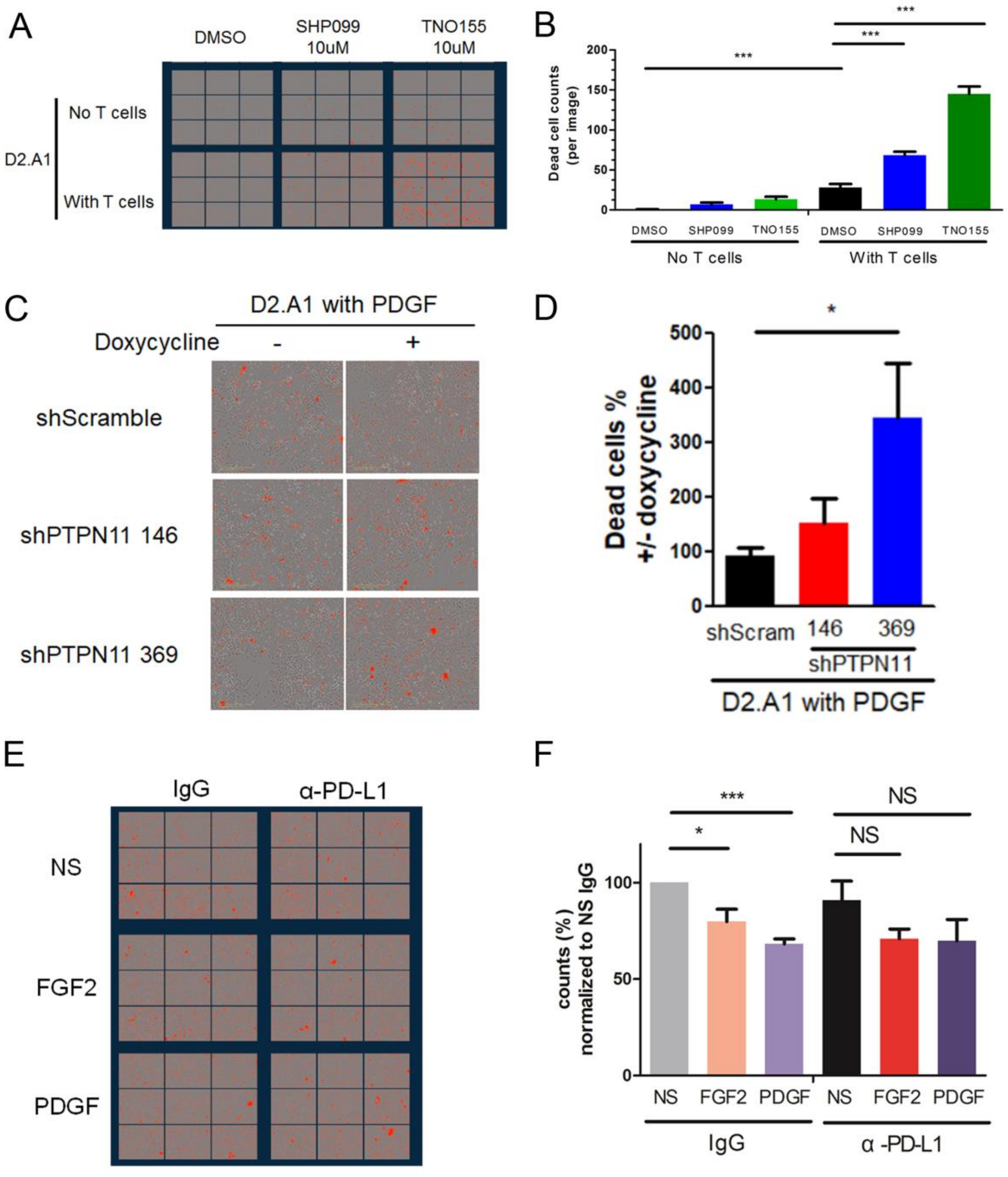

Supplementary Figure 13. Inhibition or depletion of SHP2 and PD-L1 blockade rescue T cell cytotoxicity. A, Representative images of the D2.A1 cells at 1 hour following co-culturing with T cells and treated with SHP099 or TNO155. The D2.A1 cells without T cells served as background. B, Bar graph comparing the dead cell counts per image with different treatments, ***p<0.001, n=9. C, Representative images of the D2.A1 shScramble and shPTPN11 146, 369 cells treated with/without doxycycline under the stimulation of PDGF (100ng/ml) at 1 hour following co-culturing with T cells. D, Bar graph comparing the percentage of dead cell counts with doxycycline normalized to no doxycycline. *p<0.05, n=9. E, Representative images of the D2.A1 cells treated with FGF2/PDGF and IgG/α-PD-L1 (10μg/ml) at 1 hour following co-culturing with T cells. F, Bar graph comparing the percentage of dead cell counts with different treatments normalized to No Stem (ns) IgG group. NS: not significant, *p<0.05, ***p<0.001, n=4 individual repeats.
