# Supplementary material for: Tumor Cell–Autonomous SHP2 Contributes to Immune Suppression in Metastatic Breast Cancer
Source: Cancer Res Commun. 2022 Oct 3;2(10):1104–18. doi: 10.1158/2767-9764.CRC-22-0117 (PMC10035406; doi:10.1158/2767-9764.CRC-22-0117)
Supplement: Supplementary Figure S14 — PD-L1 expression is induced by growth factor stimulation in the D2.A1 cells. [file crc-22-0117-s16.pdf]

Supplementary Figure 14

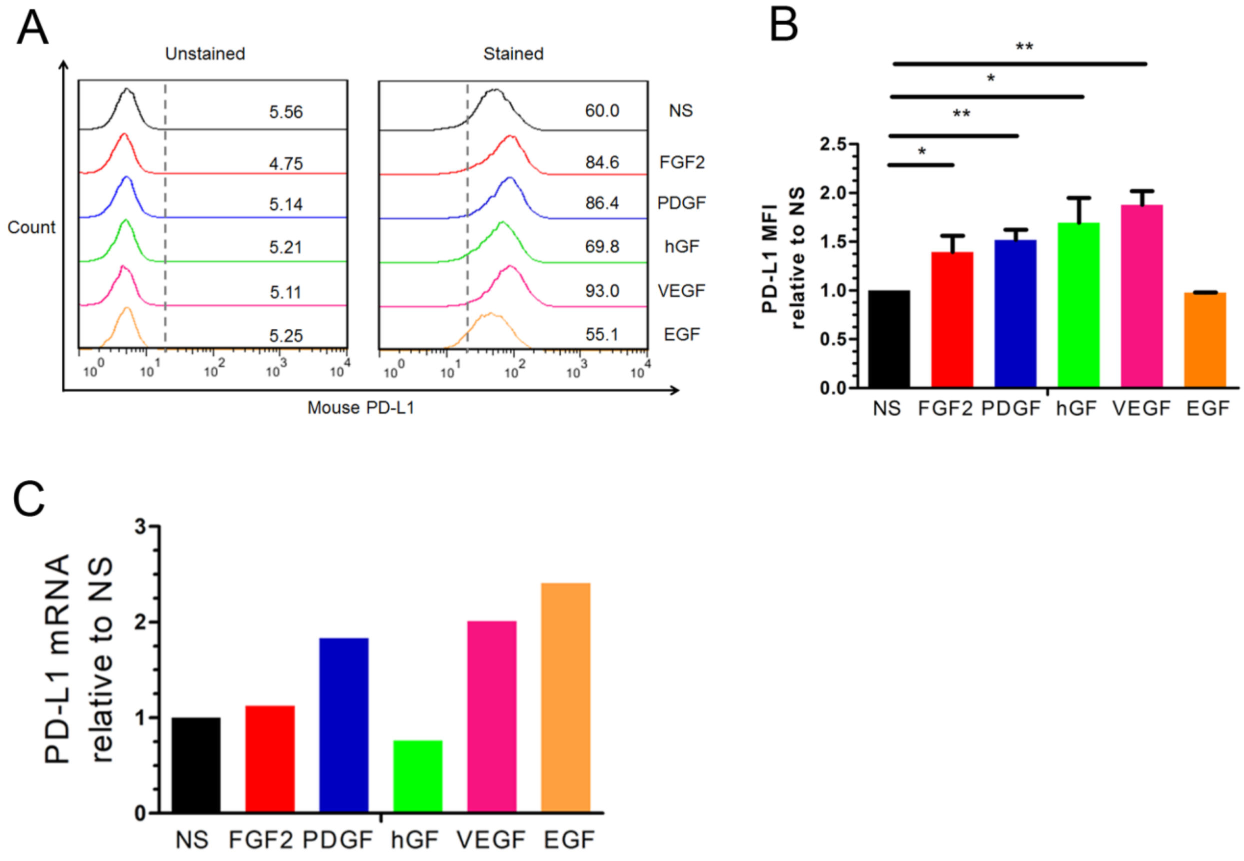

Supplementary Figure 14. PD-L1 expression is induced by growth factor stimulation in the D2.A1 cells. A, Cell surface analysis of PD-L1 in D2.A1 cells induced by different growth factors using flow cytometry. B, Bar graph comparing fold change of PD-L1 Mean Fluorescence Intensity (MFI) induced by different growth factors compared to no stimulation (NS). *p<0.05, **p<0.01, n=3. C, Bar graph comparing fold change of PD-L1 mRNA normalized to GAPDH induced by different growth factors compared to no stem (NS).
