# Supplementary material for: Tumor Cell–Autonomous SHP2 Contributes to Immune Suppression in Metastatic Breast Cancer
Source: Cancer Res Commun. 2022 Oct 3;2(10):1104–18. doi: 10.1158/2767-9764.CRC-22-0117 (PMC10035406; doi:10.1158/2767-9764.CRC-22-0117)
Supplement: Supplementary Figure S15 — Growth factors and 3D culture environment induce PD-L1 in MBC cells. [file crc-22-0117-s17.pdf]

Supplementary Figure 15

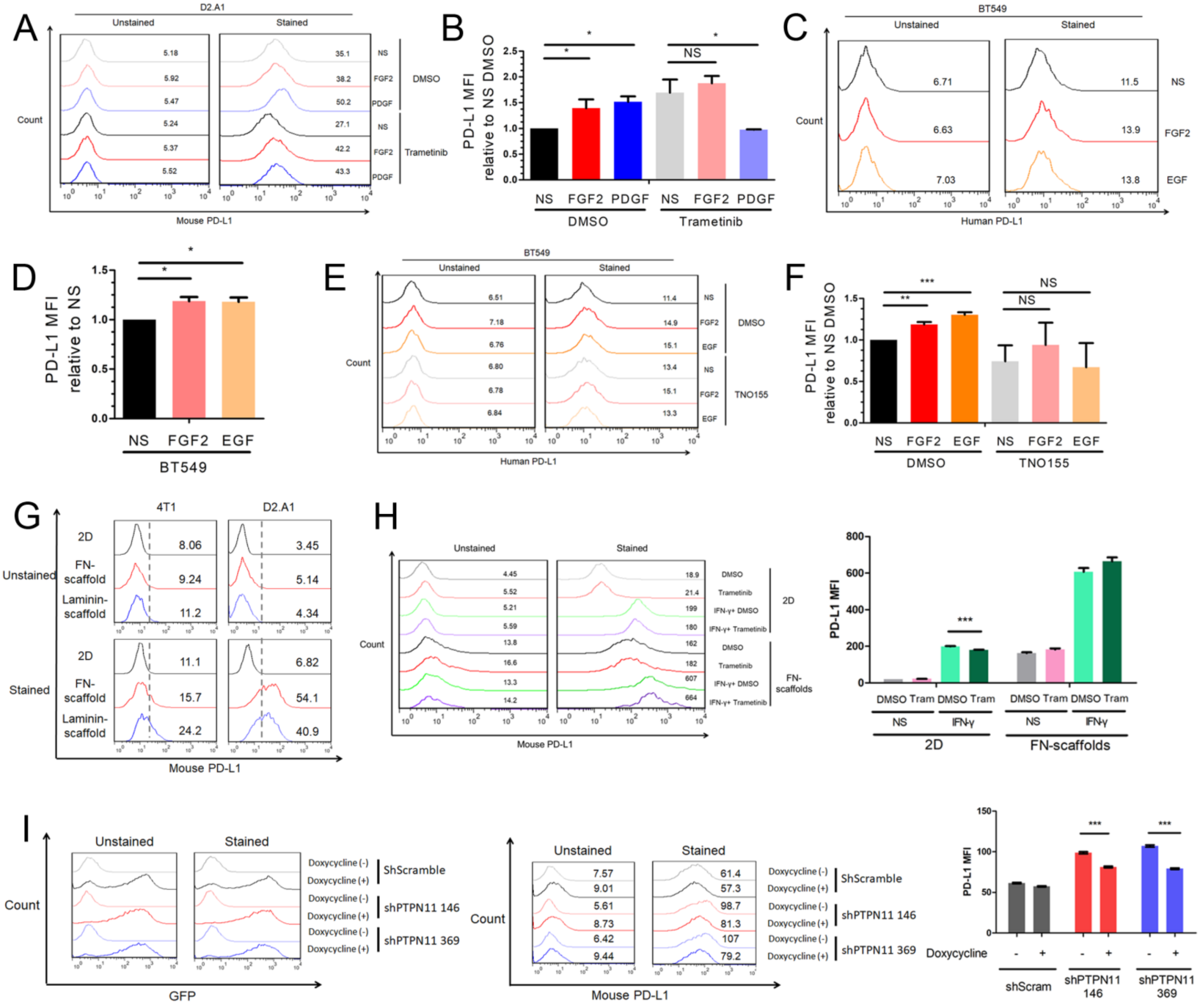

Supplementary Figure 15. Growth factors and 3D culture environment induce PD-L1 in MBC cells. A, B, Cell surface analysis of PD-L1 using flow cytometer and bar graph comparing fold change of PD-L1 Mean MFI in D2.A1 cells induced by FGF2 / PDGF and treated with Trametinib. NS: not significant,*p<0.05, n=3. C, Cell surface analysis of PD-L1 in BT549 cells induced by FGF2 and EGF using flow cytometer. D, Bar graph comparing fold change of PD-L1 Mean Fluorescence Intensity (MFI) induced by FGF2 and EGF compared to no stem (NS). *p<0.05, n=3. E, F, Cell surface analysis of PD-L1 using flow cytometer and bar graph comparing fold change of PD-L1 Mean MFI in BT549 cells induced by FGF2 / EGF and treated with TNO155. NS: not significant, **p<0.01, ***p<0.001, n=3. G, Cell surface analysis of PD-L1 using flow cytometer in 4T1 and D2.A1 cells cultured on fibronectin-coated or laminin-coated scaffolds compared to 2D culture. H, Cell surface analysis and quantification of PD-L1 using flow cytometer comparing D2.A1 cells cultured on fibronectin-coated scaffolds treated with Trametinib and interferon-γ to 2D culture. I, Cell surface analysis and quantification of eGFP and PD-L1 using flow cytometer showing the influence of cell surface PD-L1 by doxycycline inducible depletion of SHP2 in D2.A1 cells.
