# Supplementary material for: Tumor Cell–Autonomous SHP2 Contributes to Immune Suppression in Metastatic Breast Cancer
Source: Cancer Res Commun. 2022 Oct 3;2(10):1104–18. doi: 10.1158/2767-9764.CRC-22-0117 (PMC10035406; doi:10.1158/2767-9764.CRC-22-0117)
Supplement: Supplementary Figure S16 — SHP2 regulates MHC class I expression via the balance between MAPK and STAT1 signaling in human MBC cells. [file crc-22-0117-s18.pdf]

Supplementary Figure 16

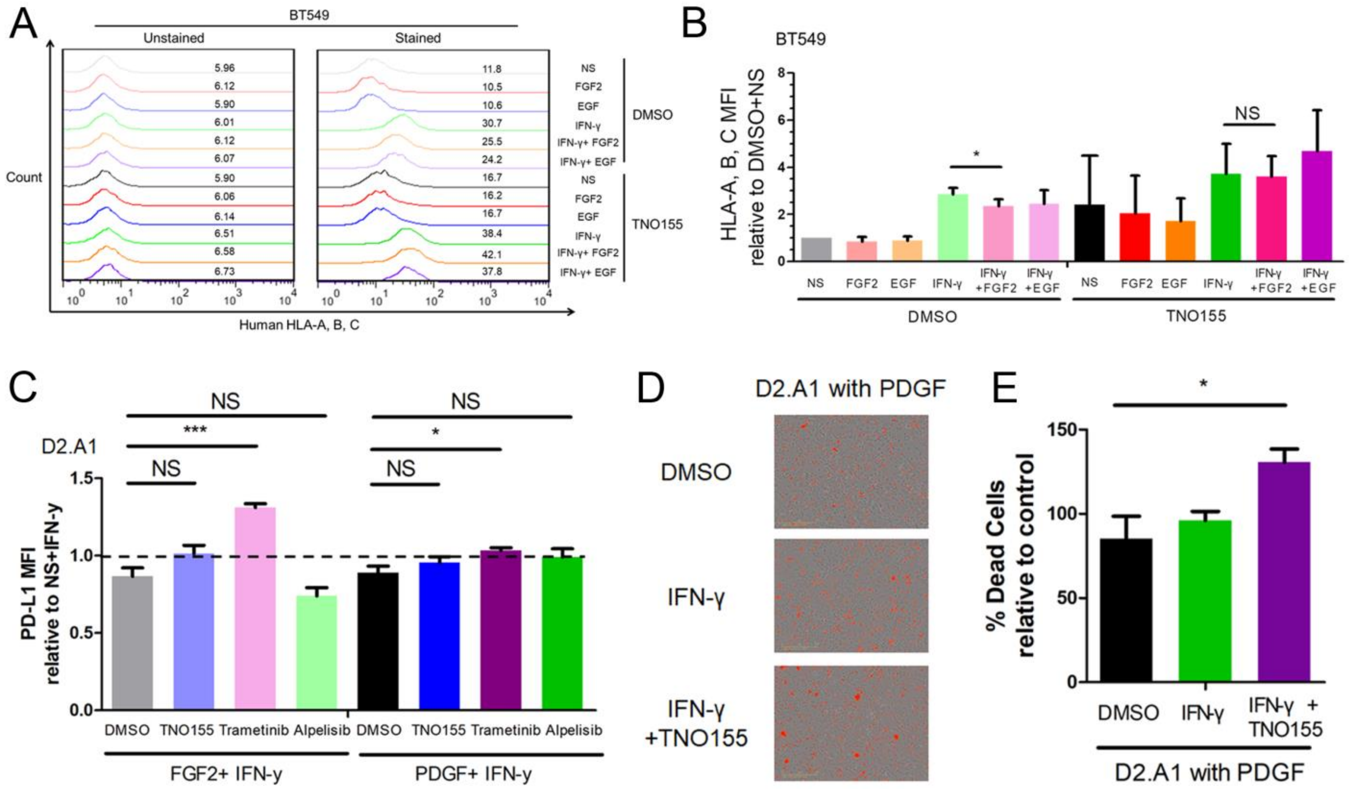

Supplementary Figure 16. SHP2 regulates MHC class I expression via the balance between MAPK and STAT1 signaling in human MBC cells. A, Cell surface analysis of HLA-A,B,C in BT549 cells treated with different growth factors, human interferon-γ (200ng/ml) and TNO155 (5μM) using flow cytometer. B, Bar graph comparing fold change of HLA-A,B,C MFI induced by different growth factors, interferon-γ and TNO155 compared to DMSO + no stem group. NS: not significant, *p<0.05, n=3. C, Bar graph comparing fold change of PD-L1 MFI induced by different growth factors and interferon-γ compared to interferon-γ alone with different inhibitors in D2.A1 cells. NS: not significant, **p<0.01, ***p<0.001, n=3. D, Representative images of the D2.A1 cells treated with interferon-γ (200ng/ml) and interferon-γ (200ng/ml) + TNO155 (5μM) under PDGF (100ng/ml) stimulation at 1 hour following co-culturing with T cells. E, Bar graph comparing the percentage of dead cell counts with different treatments normalized to control (DMSO only with no PDGF). *p<0.05, n=4 individual repeats.
